# HDAC5-mediated exosomal Maspin and miR-151a-3p as biomarkers for enhancing radiation treatment sensitivity in hepatocellular carcinoma

**DOI:** 10.1186/s40824-023-00467-7

**Published:** 2023-12-15

**Authors:** Seung Min Lee, Jeongin Cho, Sujin Choi, Dong Ha Kim, Je-Won Ryu, Inki Kim, Dong-Cheol Woo, Young Hoon Sung, Jin-Yong Jeong, In-Jeoung Baek, Chan-Gi Pack, Jin Kyung Rho, Sang-wook Lee, Chang Hoon Ha

**Affiliations:** 1 Department of Biochemistry and Molecular Biology and Asan Institute for Life Sciences, Asan Medical Center, University of Ulsan College of Medicine, 88, Olympic-Ro 43-Gil, Songpa-Gu, Seoul, 05505 Republic of Korea; 2 Department of Radiation Oncology, Asan Medical Center, University of Ulsan College of Medicine, 88, Olympic-Ro 43-Gil, Songpa-Gu, Seoul, 05505 Republic of Korea; 3 Department of Cell and Genetic Engineering, Asan Medical Center, Asan Institute for Life Sciences University of Ulsan College of Medicine, Seoul, Republic of Korea; 4 Department of Biomedical Engineering, Asan Medical Center, Asan Institute for Life Sciences, University of Ulsan College of Medicine, Seoul, Republic of Korea; 5 Department of Pharmacology, Asan Medical Center, Asan Institute for Life Sciences, University of Ulsan College of Medicine, Seoul, Republic of Korea; 6 Department of Microbiology, Asan Medical Center, Asan Institute for Life Sciences, University of Ulsan College of Medicine, Seoul, Republic of Korea; 7 Digestive Diseases Research Center, University of Ulsan College of Medicine, Seoul, 05505 Republic of Korea

**Keywords:** Exosome, MicroRNA, Maspin, Hepatocellular carcinoma, Radiotherapy

## Abstract

**Background:**

Tumor-derived exosomes are critical elements of the cell–cell communication response to various stimuli. This study aims to reveal that the histone deacetylase 5 (HDAC5) and p53 interaction upon radiation in hepatocellular carcinoma intricately regulates the secretion and composition of exosomes.

**Methods:**

We observed that HDAC5 and p53 expression were significantly increased by 2 Gy and 4 Gy radiation exposure in HCC. Normal- and radiation-derived exosomes released by HepG2 were purified to investigate the exosomal components.

**Results:**

We found that in the radiation-derived exosome, exosomal Maspin was notably increased. Maspin is known as an anti-angiogenic gene. The expression of Maspin was regulated at the cellular level by HDAC5, and it was elaborately regulated and released in the exosome. Radiation-derived exosome treatment caused significant inhibition of angiogenesis in HUVECs and mouse aortic tissues. Meanwhile, we confirmed that miR-151a-3p was significantly reduced in the radiation-derived exosome through exosomal miRNA sequencing, and three HCC-specific exosomal miRNAs were also decreased. In particular, miR-151a-3p induced an anti-apoptotic response by inhibiting p53, and it was shown to induce EMT and promote tumor growth by regulating p53-related tumor progression genes. In the HCC xenograft model, radiation-induced exosome injection significantly reduced angiogenesis and tumor size.

**Conclusions:**

Our present findings demonstrated HDAC5 is a vital gene of the p53-mediated release of exosomes resulting in tumor suppression through anti-cancer exosomal components in response to radiation. Finally, we highlight the important role of exosomal Maspin and mi-151a-3p as a biomarker in enhancing radiation treatment sensitivity.

**Graphical Abstract:**

Therapeutic potential of HDAC5 through p53-mediated exosome modulation in radiation treatment of hepatocellular carcinoma

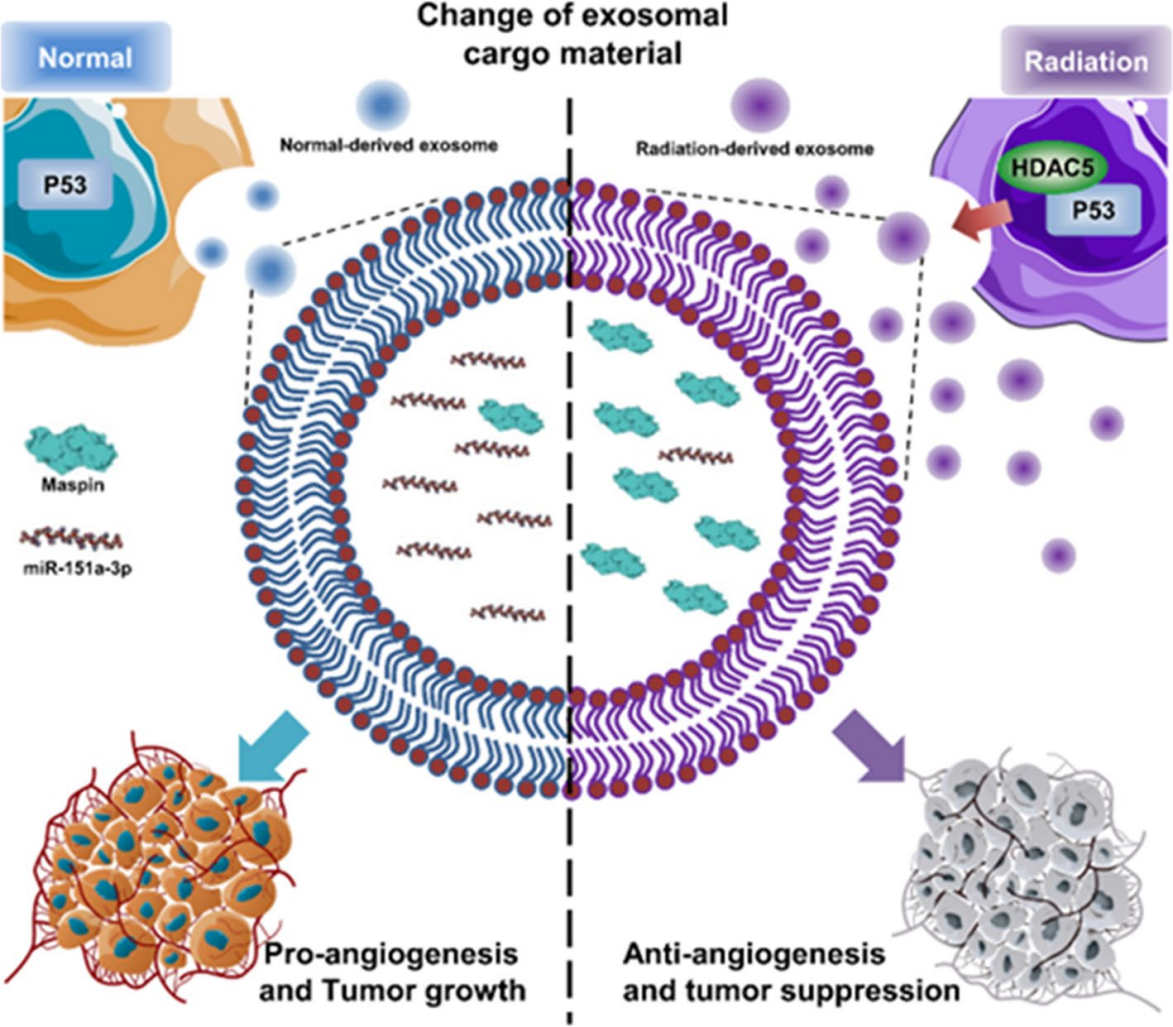

**Supplementary Information:**

The online version contains supplementary material available at 10.1186/s40824-023-00467-7.

## Introduction

Exosomes are extracellular vesicles of size 30–150 nm that are released outside the cell. A phospholipid bilayer membrane prevents exosome disruption by various enzymes. Cancer cells secrete ten times more exosomes compared to normal cells. Proteins, growth factors, chemokines, and miRNAs inside exosomes are released to regulate the microenvironment and promote the growth and communication of cancer cells [1, 2]. Post-release, tumor-derived exosomal proteins and microRNAs (miRNAs) regulate functions such as retaining growth, invasion, metastasis, and therapy resistance in tumor cells through cell–cell communication within the tumor-derived exosome. Therefore, exosomal miRNAs are crucial for mediating tumor progression and can likely be used as a promising biomarker in diagnosing and predicting cancer [3].

Histone acetylation/deacetylation plays a critical role in regulating gene expression [4, 5]. p53 modification is temporarily regulated by histone deacetylase 5 (HDAC5) [6, 7]. During post-translational modifications, the acetylation of p53 at lysine-120 of the DNA binding domain has been associated with p53-mediated apoptosis [8]. Thus, the pro-apoptotic target gene, modulated by p53, is transactivated by the k120 deacetylation of p53 by HDAC5, which determines its cellular fate as a regulator of p53 modification [7, 9]. Further, the secretion of exosomes in cells undergoing p53 response to stress is increased by effector TSAP6 downstream of p53. Therefore, exosomes can be regulated by the p53 response [10]. SerpinB5 (Maspin) is a protein regulated by the transcription factor p53 and has been shown to have anti-angiogenesis and tumor-suppressive effects [11, 12]. Additionally, upon irradiation, Maspin increased in a p53-dependent manner, was loaded onto the exosome, and released [13]. However, mechanism of HDAC5-dependent modulation of p53-related exosomal cargo upon irradiation is poorly understood.

Recently, the goal of radiation treatment (RT) has been to improve the quality of life of patients by reducing the side effects of RT. In particular, in hepatocellular carcinoma (HCC), RT has become crucial to prevent cancer progression and assist surgical treatment. In addition, RT is the most effective treatment for local tumor metastasis in patients who potentially need liver resection or transplantation [14, 15].

In this study, we present our findings on tumor-derived exosomes, including the protein- and miRNA-delivering mechanism. HDAC5 and p53 interacted upon exposure to radiation, which increased exosome release and altered the composition of exosomal proteins and miRNAs. Interestingly, the expression of exosomal Maspin was notably increased after RT, which was found to be elaborately regulated by HDAC5. The irradiated exosome with high expression of exosomal Maspin showed an exceptional anti-angiogenic response. Moreover, the levels of four miRNAs, the presence of which was inversely proportional to the survival rate of HCC patients, were reduced by RT. Among them, miR-151a-3p reduced by RT caused an increase in radioresistance and tumor growth in HCC. Compared to the HCC normal exosome, the radiation-derived exosome showed tumor growth inhibition and significantly reduced angiogenesis in the HCC xenograft model. Finally, we highlight the potential role of exosomal Maspin and miR-151a-3p as biomarkers in tumor therapeutics and RT sensitivity.

## Material and methods

### Cell culture and X-radiation treatment

HepG2 and SK-Hep1 were purchased from the American Type Culture Collection (Rockville, MD) (ATCC HTB-177). Hep3B was purchased by Korea Cellular Bank (Seoul, Korea). All cells were cultured in DMEM (Corning) with 10% fetal bovine serum, 4.5 g/L glucose, L-glutamine, sodium pyruvate, and 100 U/mL of penicillin and streptomycin, and maintained at 37 °C in a humidified chamber containing 5% CO_2_. Radiation exposure at 2 Gy and 4 Gy doses was accomplished using X-RAD320 (1 Gy/min at 320 kV, 12.5 mA, 50 cm SSD (HVL≈ 4 mm Cu)) equipment.

### Exosome purification

Radiation and non-radiation exosomes were purified using ultracentrifugation methods. 1 × 10^6^ cells were seeded in 100 mm dishes and allowed to recover overnight. Then, the cells were washed twice with pre-warmed PBS, and the culture medium was replaced with exosome-free medium supplemented with 5% exosome-depleted FBS (SBI). The radiation exosomes were irradiated with 4 Gy radiation using X-RAD 320 (1 Gy/min at 320 kV, 12.5 mA, 50 cm SSD (HVL≈ 4 mm Cu)) equipment. The medium was subjected to gradient centrifugation. Briefly, the medium was first centrifuged at 1000 × g for 10 min, then at 3000 × g for 30 min, and finally centrifuged in a Beckman Coulter Optima™ L-XP Ultracentrifuge System at 100,000 gavg at 4℃ for 90 min with a SW 28 Swinging-Bucket Rotor (k-factor: 71) to pellet exosomes. The supernatant was carefully removed, and crude exosome-containing pellets were resuspended in 1 mL of ice-cold PBS and pooled. A second round of ultracentrifugation [100,000 gavg at 4℃ for 90 min with a Type SW 28 Swinging-Bucket Rotor (k-factor: 71)] was performed, and the resulting exosome pellet was resuspended in 500 μL of PBS.

### Western blot

The cells were collected, washed with PBS, and lysed in lysis buffer containing protease inhibitors (GenDEPOT, USA). After determining the protein concentration with a Bradford Protein Assay Reagent (Bio-Rad), equal amounts of protein were separated on 10% SDS-PAGE, electrically transferred to nitrocellulose membrane, and blocked with 5% skim milk and 5% BSA. The membranes were incubated with anti-HDAC5 (1:1000; Cell Signaling Technology, USA), anti-p53 (1:2000; Santacruz, USA), anti-p21 (1:2000; Cell Signaling Technology), anti-Puma (1:2000; Cell Signaling Technology), anti-STEAP3 (1:1000; Proteintech, USA), anti-Maspin (1:1000; Cell Signaling Technology), anti-CD63 (1:1000; Santacruz), anti- CD81 (1:1000; Santacruz), anti-HSP90 (1:1000; BD bioscience), anti-BCL2 (1:1000; Cell Signaling Technology), anti-E-cadherin (1:1000; Agilent Dako), anti-Twist1 (1:1000; Abcam), N-cadherin (1:1000; Cell Signaling Technology), Vimentin (1:1000; Calbiochem), or anti-beta-actin (1:1000; Santacruz) primary antibody at 4 °C with overnight shaking. After washing twice, the membranes were incubated with horseradish peroxidase (HRP)-conjugated anti-rabbit and anti-mouse secondary antibody (Jackson ImmunoResearch Inc. USA) at room temperature for 1 h. Finally, the membranes were incubated with WESTSAVE-UP western blotting substrate (Youngin frontier, Korea), and images were visualized using ChemiDoc imaging system (Bio-Rad, USA).

### Duolink proximity ligation assay (PLA)

The DuoLink® In Situ Red Starter Kit Mouse/Rabbit (DUO92101, Sigma-Aldrich, Darmstadt, Germany) was used to detect interacting target proteins. Cells were seeded in eight-well chamber removable slides (Ibidi GmbH Am Klopferspitz, Germany) and cultured for 24 h. Then, HepG2 cells were exposed to radiation (4 Gy), and after 12 h, the slides were washed with cold 1xPBS and fixed in 4% paraformaldehyde for 30 min. Subsequently, slides were blocked with Duolink Blocking Solution in a pre-heated humidified chamber for 30 min at 37 °C. The primary antibody to detect HDAC5 and p53 was added to the slides and incubated overnight at 4 °C. Then, slides were washed with 1 × wash buffer A and subsequently incubated with the two PLA probes (1:5 diluted in antibody diluents) for 1 h, followed by incubation with the Ligation-Ligase solution for 30 min and the Amplification-Polymerase solution for 100 min in a pre-heated humidified chamber at 37 °C. Before imaging, slides were washed with 1 × wash buffer B and mounted with a cover slip using Duolink In Situ Mounting Medium with DAPI. Fluorescence images were acquired using a Zeiss LSM 780 confocal microscope [16].

### Co-immunoprecipitation

HepG2 were treated with or without 4 Gy radiation and incubated for 12 h. HepG2 were lysed with lysis buffer (25 mM Tris–HCl pH 7.5, 150 mM NaCl, 1 mM EDTA pH 8.0, triton X-100 0.01%, PMSF 1 mM, 1 × phosphatase inhibitor cocktail) and incubated with rotation at 4 °C for 1 h. Thereafter, the whole lysate immunoprecipitation was performed according to standard co-immunoprecipitation procedure [17]. Briefly, whole lysates were pre-cleared with Protein A/G-agarose beads (sc-2003; Santa Cruz Biotechnology) at 4 °C for 1 h. Afterward, normal IgG and p53 antibodies (2 μg) were added and incubated with rotation at 4 °C for 24 h. The next day, 20 μL of Protein A/G-agarose beads were added and incubated with rotation at 4 °C for 1 h. Protein A/G-agarose beads were separated by centrifugation and washed. Precipitated proteins were resolved in 2 × SDS buffer, and western blot was performed. Precipitated proteins prepared using anti-p53 antibody were co-immunoprecipitated with anti-HDAC5 antibody.

### Transmission electron microscopy (TEM)

To photograph exosomes, 0.1 M of exosome pellets was fixed in 2.5% glutaraldehyde in 0.1 M cacodylate solution (pH 7.0) for 1 h and then fixed in 2% osmium tetroxide for another hour (4 °C). After dehydration using the graded acetone series, embedding was performed using Spurr's medium (Electron Microscopy Sciences). The resulting section sample was cut at 60 nm with an ultramicrotome (RMC MTXL, USA), followed by double staining using 2% uranyl acetate for 20 min and citrate for 10 min. Prepared sections were photographed at 80 kV using Hitachi H-7600 TEM (Hitachi, Japan) equipment.

### Exosome size distribution and concentration measurement

To determine exosome size distribution and concentration, nanoparticle tracking-based analyses were performed using a NanoSight (NS500) apparatus (Malvern Instruments Ltd.). Samples were diluted to provide counts within the linear range of the instrument. Videos of 1-min duration were recorded for each sample, with a frame rate of 30 frames per second. Particle movement was analyzed using NTA software (NTA 2.3; NanoSight Ltd.) according to the manufacturer's protocol. The NTA software was optimized to first identify and then track each particle on a frame-by-frame basis [18, 19].

### Transient transfection

Cells were seeded in 6-well-plates at 10^5^ cells/mL/well before transfection and radiation. On the following day, HepG2 cells were transfected when the cells reached approximately 80% confluence and were exposed to 4 Gy radiation using X-RAD 320. miRCURY LNA miR-151a-3p mimic (miRCURY LNA miRNA Mimic, MIMAT0000757) was purchased from QiAGEN (Qiagen Korea Ltd.). Pre-designed HDAC5 siRNA and Scrambled siRNA / miRNA controls were purchased from BIONEER (Seoul). The final concentrations of miRNA were 50–100 nM and siRNA were 100 nM. Transfections were conducted with Lipofectamine 3000 (Invitrogen) according to the manufacturer’s instructions.

### Quantitative real time reverse transcription polymerase chain reaction(qRT-PCR)

Total RNA was extracted using a miRNeasy Mini Kit (Qiagen Korea Ltd.) according to the manufacturers' protocols. To quantify matured cellular and exosomal miRNAs, the extracted total RNA was polyadenylated with a poly(A) tailing kit (Ambion, Austin, TX) prior to reverse transcription. Reverse transcription was performed using cDNA using PrimeScript™ Reverse Transcriptase (Takara Bio Inc.). The cDNA samples were used for quantitative RT-PCR analysis in triplicate to determine miR-151a-3p expression levels using a TB Green® Premix Ex Taq™ (Takara Bio Inc.) and CFX96 Detection System real-time PCR instrument.

### In-vitro angiogenesis assay

For the endothelial cell tube forming assay, growth factor–reduced Matrigel (354230, Corning) was used in a 24-well plate, and 100 μl of each well was added and coated. After incubation at 37 °C for 30 min, HUVECs were seeded at 3 × 10^4^ cells per well. recombinant Maspin was treated with 1–10 µg/ml and normal- and RT-induced exosome was treated with 30 µg/ml. After incubation for 16 h, the cells were stained with calcein-AM dye (1 μg/ml) and photographed using EVO M5000 Imaging System (Invitrogen). The tube organization of each well was counted and analyzed by fold increase compared with a control sample.

### Ex-vivo aorta ring assay

The mouse aorta ring assay was performed as described previously. Each well of a 24-well plate was coated with 120 μl Matrigel (Corning) and polymerized in an incubator. Aortas isolated form 8- to 10-week-old male C57/BL6 mice were cleaned of periadventitial fat and connective tissues in cold PBS and then cut into rings with circumferences of 1 mm. The aortic rings were randomly placed into wells. 400 μl of serum-free medium 200, with normal exosome (30 µg/ml) or radiation-derived exosome (30 µg/ml), was added to each well. The medium was exchanged every 2 days. After 10 days, the aortic rings were stained with 1 mM calcein (AM) (Invitrogen). fluorescence images were photographed using EVO M5000 Imaging System (Invitrogen).

### Proliferation assay

The cell proliferation assay was performed using the Chromo-CK Cell Viability Assay KIT (Monobio Seoul) according to the manufacturer’s instructions. Transfected cells were harvested at the designated times after seeding. Briefly, the reagent (10 μL/well) was added to 100 μL of medium containing cells in each well of a 96-well plate and incubated for 1 h at 37℃ under humidified 5% CO_2_ in air. For colorimetric analysis, absorbance at 450 nm was recorded using an ELx800 Absorbance Microplate Reader (SpectraMax 340PC Microplate Reader, Molecular Devices, USA). Each experiment was repeated at least thrice.

### Migration and invasion assay

Tumor cell migration and invasion were analyzed in 24-well plates with 8-μm pore size polycarbonate membranes (BD, NJ, USA). For invasion assays, the membranes were coated with diluted Matrigel (BD, NJ, USA) to form matrix barriers. HepG2 cells were transfected with 151a-3p or negative control. For the migration and invasion assays, the cells (5 × 10^4^ for migration, 1 × 10^5^ for invasion) were resuspended in 200 μL of serum-free DMEM at 24 h post-transfection and added to upper compartments, and lower compartments were filled with 600 μL of DMEM with 10% FBS (different substrates were added accordingly). After incubation at 37 °C for 10 h (migration) or 24 h (invasion), the cells remaining on the upper surfaces of the membrane were removed. The cells on the lower surfaces of the membrane were fixed, stained with crystal violet, and counted under a light microscope. Each experiment was repeated at least thrice.

### Cell cycle analysis

Transfected cells were collected and resuspended after radiation exposure. Annexin V-fluorescein isothiocyanate (FITC) and propidium iodide (PI) staining assays were conducted to detect the percentage of apoptotic (FITC-stained) and necrotic (PI-stained) cells in a given population. Analyses were performed using a FACS canto flow cytometer (BD Biosciences, Franklin Lakes, NJ) according to the manufacturer’s instructions. The experiments were performed in triplicate [20].

### Apoptosis

Transfected cells were collected and resuspended in cold PBS after radiation exposure, and fixed in cold 70% ethanol added dropwise to the cell pellet while vortexing. They were then stored at -20℃ overnight. Cells were rehydrated with PBS for 10 min at RT and subsequently stained with PI staining solution containing 50 μg/mL PI (BD Biosciences, Franklin Lakes, NJ, USA) and 100 μg/mL RNase A, DNase, and protease-free (Thermo Scientific) in 1X binding buffer. Analyses were performed using a FACS canto flow cytometer (BD Biosciences, Franklin Lakes, NJ, USA) according to the manufacturer’s instructions. The experiments were performed in triplicate.

### Immunohistochemistry

For histological analysis, xenograft-derived tumor tissue was fixed in 4% paraformaldehyde for 24 h. It was embedded in a paraffin block and sectioned at 5 μm thick. Anti-CD31 antibody (abcam, Cambridge, UK) was used to evaluate the capillary density infiltrated within the tumor. Afterward, the process was carried out using the VECTASTAIN ABC kit and following the manual provided by the manufacturer. 3,3′-Diaminobenzidine (DAB) Enhanced Liquid Substrate System tetrahydrochloride (Sigma, USA) was used for visualization. After counterstaining with hematoxylin, it was mounted and observed under a histological microscope (olympus BX53, Japan).

### Subcutaneous tumor xenograft models

BALB/c nude mice (5-week-old male) were purchased from Charles River Laboratories, Inc (Wilmington, Massachusetts) and fed normal rodent laboratory animal feed. HepG2 cells (5 × 10^6^) in 100 µL were injected into the right flank of a 5-week-old nude mouse in a 1:1 ratio with Matrigel (corning 356,234). Cells were treated with invivo-jetPEI/target miRNA complex after the average size of the transplanted solid cancer reached more than 100 mm^3^. Invivo-jetPEI was used to efficiently deliver miRNA; it was mixed with 5% glucose solution at a ratio of miRNA (10 µg)/invivo-jetPEI (1.2 µL) and administered in a total dose of 100 µL, thrice every 3 days via intratumoral injection. In the exosome treatment HCC xenograft model, after the average size of the transplanted solid cancer reached more than 200 mm^3^, HepG2 derived normal- and RT-induced exosome (20 µg/each tumor) was intratumorally injected 3 times at 3-day intervals. Tumor size was measured using a standard ABS digimatic caliper (CD-15AX) every 3 days after transplantation. Tumor volume was measured using the following standard formula (length × width2 × 0.52). When the volume of the largest tumor of the implanted nude mice in the experimental group exceeded 1000 mm^3^, the tumor was separated after sacrifice, its weight measured, and target protein expression in cancer tissue was analyzed.

### Statistical analysis

All experiments were performed thrice. Mathematical data are expressed as mean ± S.E.M. Unless otherwise indicated, the differences between two groups were analyzed using a Student's t-test (two-tailed). Significant numerical differences between the two groups were analyzed using Student's t-test (two-tailed), unless stated otherwise. The overall survival curve was plotted using the Kaplan–Meier method and compared using the log-rank test. Differences were considered statistically significant at *P* < 0.05. All statistical analysis was performed using SPSS13.0 software (SPSS, Chicago, IL, USA).

## Results

### Modulation of p53 and HDAC5 by RT

**Fig. 1:**
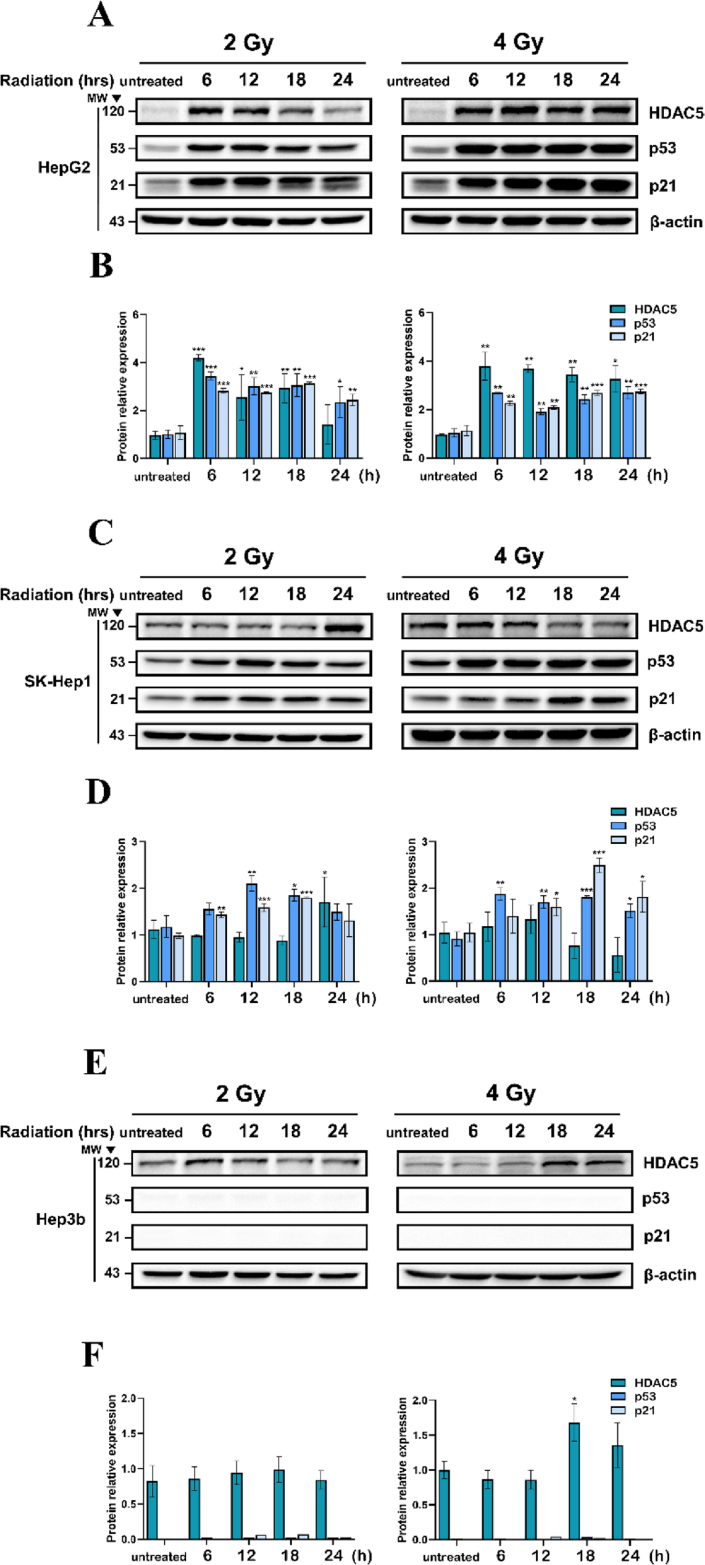
Modulation of protein p53 and HDAC5 expression in HCC after RT. Following radiation exposure at 2 Gy and 4 Gy, protein expression of p53, P21, and HDAC5 were confirmed by western blot in a time-dependent manner up to 24 h per 6 h (**A-F**). The expression of each protein was quantified based on b-actin levels. Data are shown as mean ± SEM of three independent experiments. *P < 0.05, **P < 0.01, and ***P < 0.001 vs. untreated by Student’s t-test

The expression of HDAC5 and p53 can be increased by DNA damage, and p53 is known to be increased by radiation in various cancers. To verify that protein p53 and HDAC5 were modulated by radiation, HCC cells were treated with radiation doses of 2 Gy and 4 Gy and HDAC5 and p53 expression was observed. HepG2 and SK-Hep1 are p53 wild types (p53^+/+^) while Hep3B is p53 deletion type (p53^−/−^). Individual protein expression was measured using western blot at 6, 12, 18, and 24 h (Fig. 1A-F). In HepG2, HDAC5 and p53 expression was significantly increased from 6 h after irradiation at 2 Gy and 4 Gy compared with that in the control (Fig. 1A, B). In SK-Hep1, HDAC5 expression increased after 2 Gy irradiation. However, after 4 Gy irradiation, it was reduced compared with that in the control (Fig. 1C, D). Hep3B showed an independent increase in HDAC5 expression without p53 after irradiation (Fig. 1E, F). Overall, the expression of HDAC5 and p53 can be increased by RT in various hepatocellular carcinoma cell lines, and HepG2 showed the most significant increase in expression.

### Regulation of p53 and p53-dependent exosome secretion pathway by RT-induced HDAC5

**Fig. 2:**
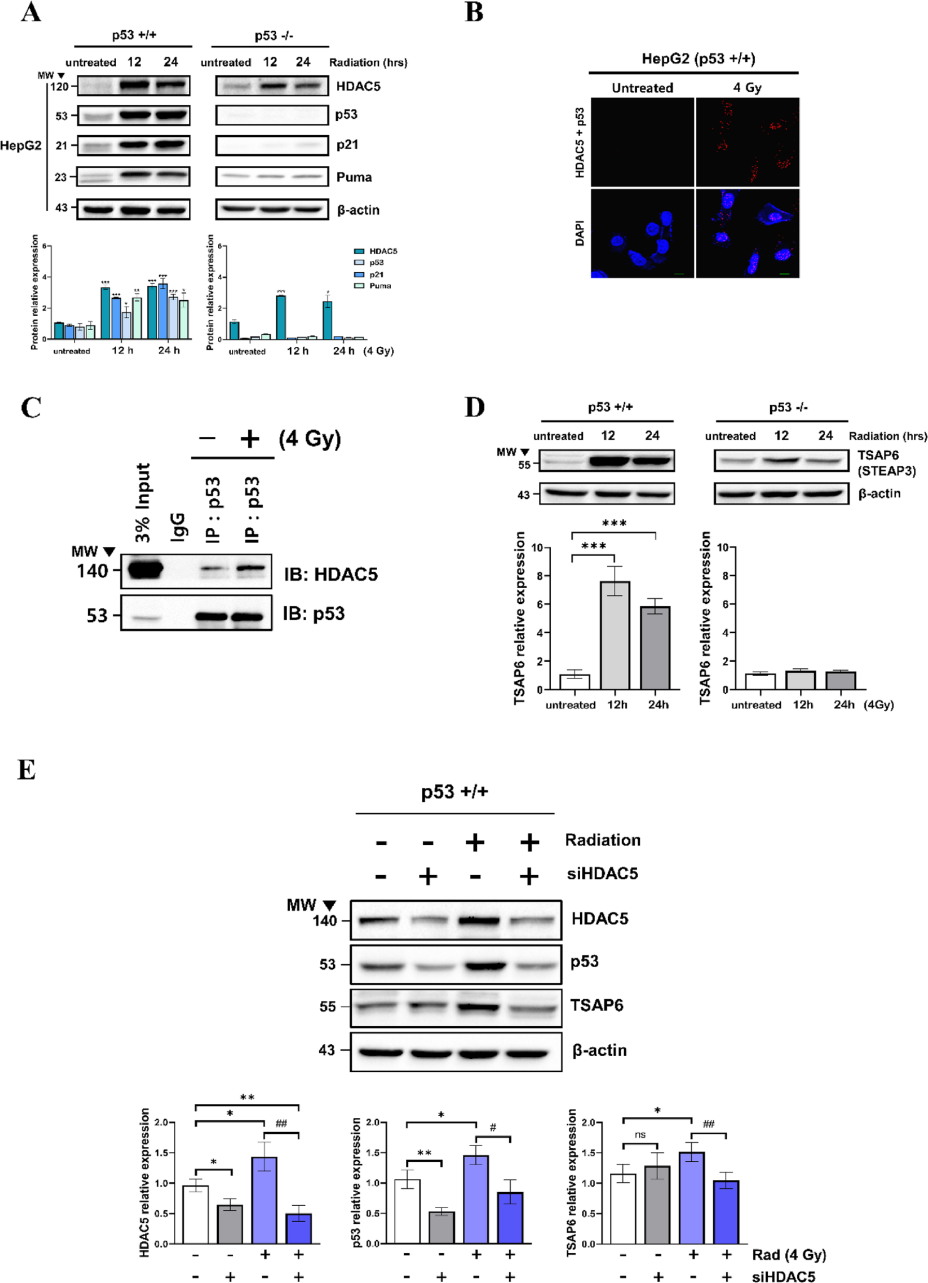
Interaction between HDAC5 and p53 induced by RT and activation of HDAC5 mediated p53-dependent exosome secretion pathway in HCC. RT-induced expression of HDAC5, p53, P21, and PUMA in p53^−/−^ and p53^**+/+**^ HepG2 was confirmed by western blot and quantified using b-actin as an internal control (**A**). Data are shown as mean ± SEM of three independent experiments. *P < 0.05, **P < 0.01, and ***P < 0.001 vs. untreated by Student’s t-test. The interaction between HDAC5 and p53 after RT was confirmed using Duolink proximity ligation assay in HepG2 (**B**). p53^**+/+**^ HepG2 cells were treated with 4 Gy radiation and incubated for 12 h, and were then immunoprecipitated with anti-p53 antibodies. Precipitated proteins were subjected to co-immunoprecipitation (CO-IP) using an anti-HDAC5 antibody and analyzed by immunoblotting (**C**). The expression of TSAP6 induced by RT in p53^−/−^ and p53^**+/+**^ HepG2 was verified by western blot. TSAP6 expression was quantified using b-actin as an internal control (**D**). Data are shown as mean ± SEM of three independent experiments. *P < 0.05, **P < 0.01, and ***P < 0.001 vs. untreated by Student’s t-test. Twenty-four hours after siHDAC5 was transfected into HepG2, exposed to 4 Gy radiation. After 12 h incubation, HDAC5, p53, and TSAP6 expressions were confirmed by western blot and quantified using b-actin as an internal control (**E**). Data are shown as mean ± SEM of three independent experiments. *P < 0.05, **P < 0.01, and ***P < 0.001 vs. untreated by Student’s t-test; #*P* < 0.05, ##*P* < 0.01, and ###*P* < 0.001 vs. 4 Gy treatment by Student’s t-test

To study the relationship between HDAC5 and p53, a p53-/- HepG2 cell line was created using the CRISPR CAS9 system (Fig. S1). p53^+/+^ and p53^−/−^cells were exposed to radiation and the expression of HDAC5 and p53, p21, Puma was measured using western blot. HDAC5 expression was increased by irradiation irrespective of intracellular p53 expression, and the relationship between HDAC5 and p53 was identified (Fig. 2A). Using FACS, cell cycle arrest and apoptosis were observed in p53^+/+^ and p53^−/−^cells after 4 Gy irradiation (Fig. S2-3) [8, 16]. The Duolink PLA assay was used to identify the interaction between p53 and HDAC5 more accurately. Radiation exposure substantially increased intracellular p53 and HDAC5 interaction (Fig. 2B). In addition, the binding of HDAC5 and p53 was significantly increased by 4 Gy radiation in CO-IP (Co-immunoprecipitation) (Fig. 2C). After exposure to 4 Gy radiation, the endogenous expression of cellular HDAC5 and p53 was confirmed to be increased in a time-dependent manner by ICC (Immunocytochemistry) in HepG2 (Fig. S4). We validated whether TSAP6 (Tumor Suppressor Activation Pathway 6), an exosome-modulated protein directly downstream of and controlled by p53, is increased by RT [10, 13]. TSAP6 expression was increased by radiation and maintained in a p53-dependent manner (Fig. 2D). We hypothesized p53-dependent exosome secretion pathway might be regulated by HDAC5. HDAC5 siRNA transfection and co-treatment with RT confirmed that p53 and TSAP6 are regulated by HDAC5. RT-induced overexpression of p53 and TSAP6 was significantly suppressed by HDAC5 knockdown (Fig. 2E). Collectively, HDAC5 is expressed p53-independently, and RT increases the interaction between HDAC5 and p53. HDAC5 functions as an upstream regulator manipulating the RT-induced p53-dependent exosome secretion pathway.

### Exosome secretion in a p53-dependent manner by RT

**Fig. 3:**
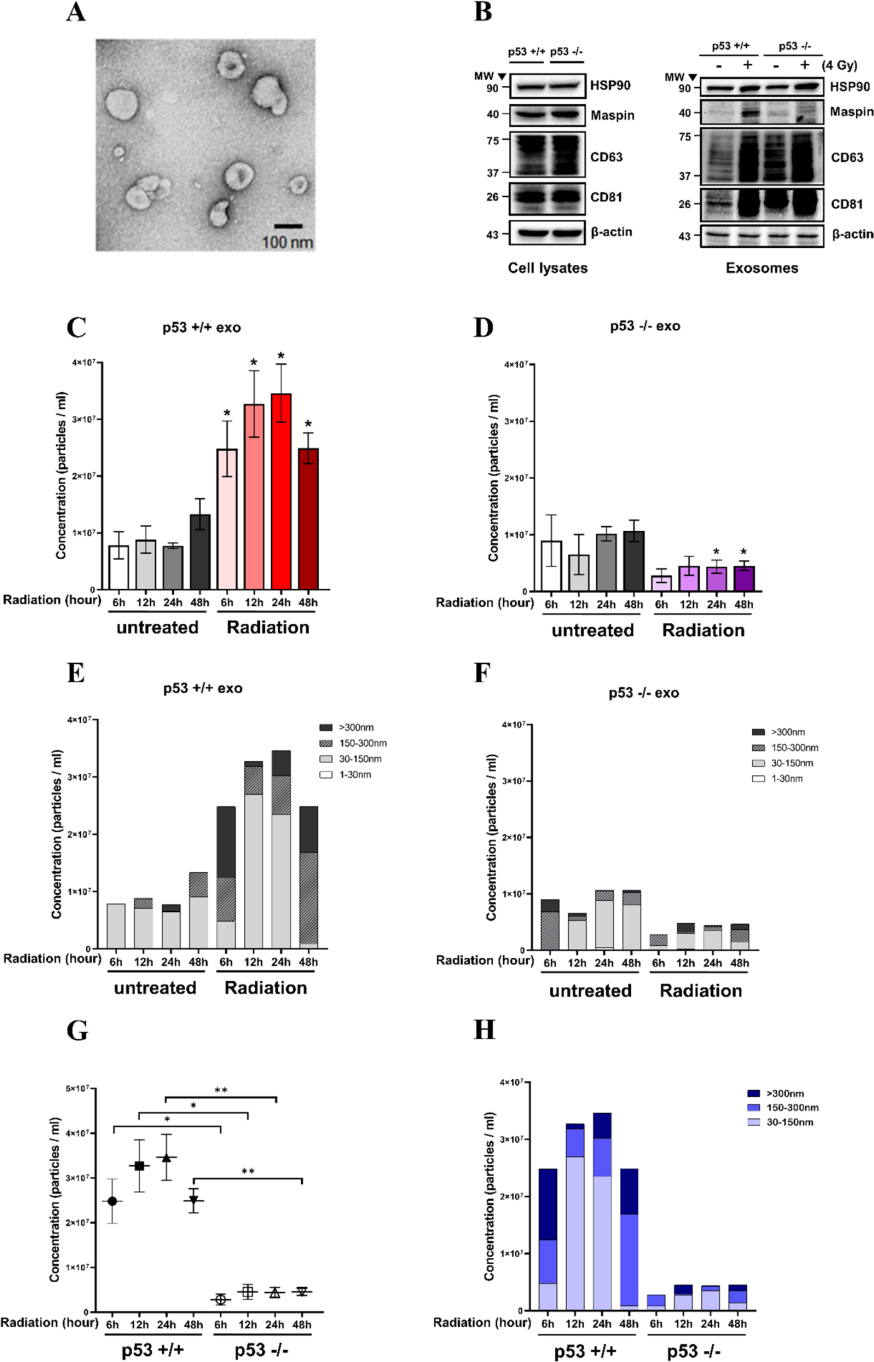
Time-dependent measurement and identification of the difference in exosome size and release amount determined by p53 gene in HCC. Electron microscopy was used to image resuspended exosome particles isolated from HepG2 (**A**). The expression of exosome marker proteins HSP90, CD63, CD81, and Maspin in each sample (10 µg) after radiation exposure was verified using western blot (**B**). Time-dependent measurements were made to determine whether the secretion of extracellular vesicles (EV) changes after radiation exposure using Nanosight's Nanoparticle Tracking Analysis (NTA) (**C**, **D**). Data are shown as mean ± SEM of three independent experiments. *P < 0.05 vs. each time of control by Student’s t-test. Based on the analyzed nanosight data, three ranges (30–150 nm, 150–300 nm, > 300 nm) were specified based on vesicle size (**E**, **F**). The secretion of EVs was analyzed qualitatively and quantitatively for the presence or absence of Tp53 genes after radiation exposure (**G**, **H**). Data are shown as mean ± SEM of three independent experiments. *P < 0.05, **P < 0.01 vs. each time of WT by Student’s t-test

Exosome secretion is released through a p53-dependent pathway. We validated exosomes released in an RT-induced p53-dependent pathway. The characteristics of exosomes derived from p53^+/+^, p53^−/−^cells were identified. Exosomes secreted by HepG2 were observed using an transmission electron microscope (TEM) (Fig. 3A). Western blot analysis showed that microvesicles released from p53^+/+^ and p53^−/−^cells expressed exosome markers CD63, CD81, and HSP90. All exosome markers have been demonstrated [18] (Fig. 3B). We also identified the discriminating expression of Maspin (SerpinB5), which is released in a p53-dependent manner from exosome upon radiation exposure [13]. Inside p53^+/+^cell-derived exosomes, Maspin expression was increased by radiation. However, p53^−/−^cell-derived exosomes did not show Maspin expression upon radiation exposure (Fig. 3B). p53 directly binds to the p53 gene-consensus binding site in the Maspin promoter by activating the Maspin promoter and inducing Maspin expression [11]. We confirmed that exosomal Maspin was released in a p53-dependent manner by RT-induction. Exosomes are secreted by an RT-induced p53-dependent pathway. We quantitatively and qualitatively analyzed the amount of exosome release from p53 + / + and p53-/- cells. Experiments were conducted at 6, 12, 24, and 48 h intervals after exposure to 4 Gy radiation, and exosomes released at each hour were extracted using ultracentrifugation. The qualitative and quantitative comparison and analysis of the isolated exosomes were performed using Nanosight NT300 (Fig. 3 & S6). The volume of p53^+/+^cell-secreted vesicles increased more than twice when exposed to radiation (Fig. 3C). Conversely, p53^−/−^cells exhibited a decrease in the volume of vesicle secretion after radiation exposure (Fig. 3D). The secreted extracellular vesicles were analyzed qualitatively by separating them into size ranges of 30–150, 150–300, and > 300 nm. The p53^+/+^cell-secreted exosome size fraction (30–150 nm) showed the most significant increase of 12 h after radiation exposure (Fig. 3E). Conversely, p53^−/−^cell-derived exosome-sized vesicles released after irradiation were rapidly reduced (Fig. 3F). The exosomes of p53^+/+^ and p53^−/−^cells released after irradiation were analyzed qualitatively and quantitatively (Fig. 3G, H). Exosomes of 30–150 nm size increased or decreased in a p53- dependent manner.

### Sophisticated regulation of RT-induced HDAC5-dependent Maspin expression and anti-angiogenic function of Maspin

**Fig. 4:**
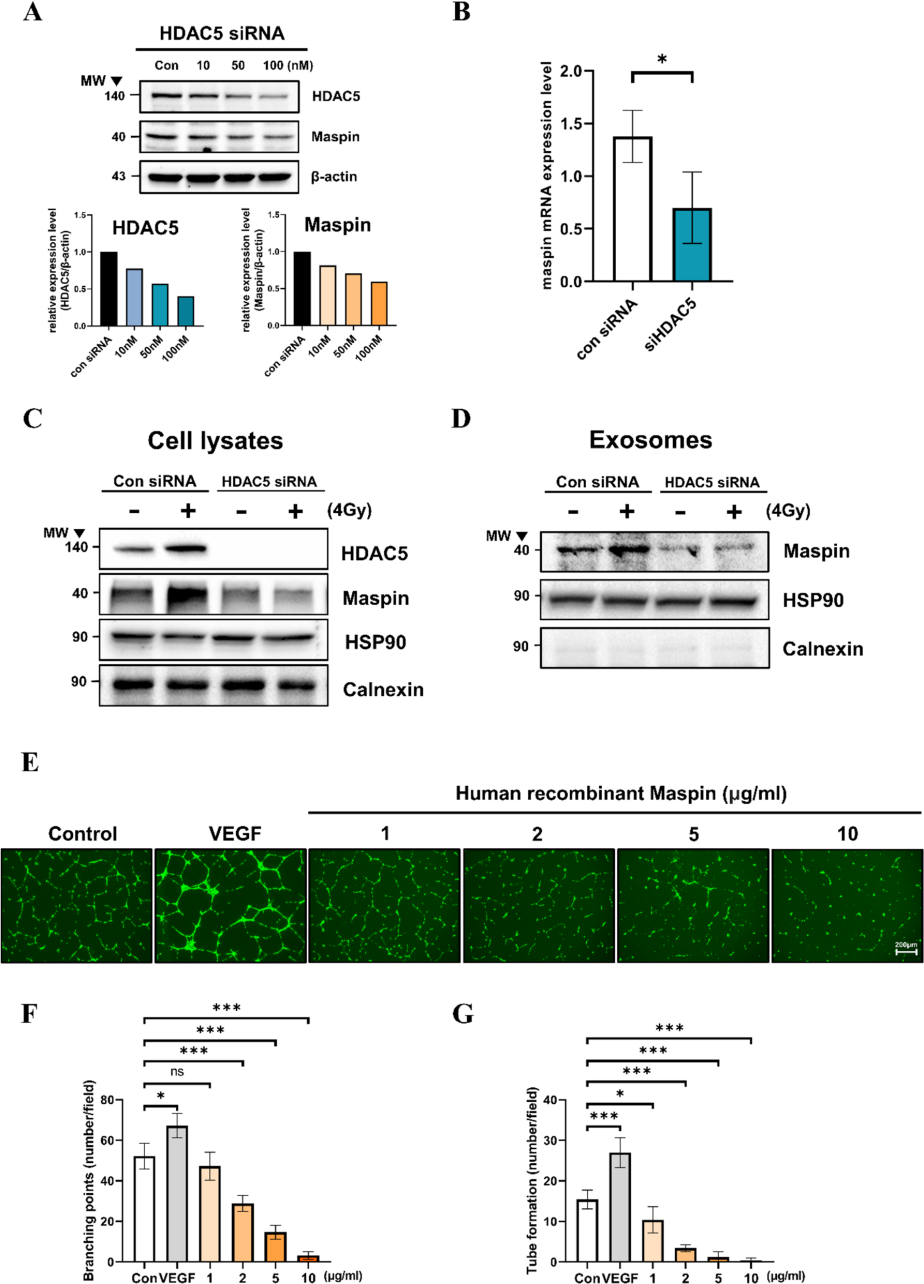
Expression of upstream regulator HDAC5-dependently regulated cellular and exosomal Maspin and anti-angiogenic function caused by human recombinant Maspin. HDAC5 siRNA was transfected into HepG2 in a dose-dependent manner, and after 24 h, HDAC5, p53, Maspin, and TSAP6 expressions were analyzed by western blot. The expression of each protein was quantified using b-actin as an internal control (**A**). After HDAC5 siRNA was transfected into HUVECs, Maspin mRNA expression was investigated. It was confirmed using the GSE15499 GEO data set (**B**). Data are shown as mean ± SD of three independent values. *P < 0.05 vs. con siRNA by Student’s t-test. HepG2 was exposed to 4 Gy RT 24 h after HDAC5 siRNA was transfected. After overnight, the expression of HDAC5, Maspin, HSP90, and Calnexin in cell lysates and exosomes of each group was examined by western blot (**C**, **D**). HUVECs were seeded on growth factor-reduced matrigel-coated plates and treated with VEGF (50 ng/ml), human recombinant Maspin (1-10ug/ml). After 16 h, calcein am dye (0.5ug/ml) was treated and fluorescence images were photographed using EVO M5000 Imaging System (Invitrogen) (**E**). Five locations of each well were randomly photographed, and the branching and tube organization were measured and compared with that of the control condition (**F**, **G**). Cell culture dishes were performed in at least three separate experiments. Scale bar = 200 μm. Data are shown as mean ± SEM. *P < 0.05, **P < 0.01, and ***P < 0.001 vs. control by Student’s t-test

Post-RT, exosomal Maspin expression was found to be increased, and it was released in a p53-dependent manner (Fig. 2F). We aimed to determine whether HDAC5 could regulate the p53-dependent expression of Maspin. HDAC5 siRNA transfection in HepG2 was performed in a dose-dependent manner. Cellular Maspin was reduced by HDAC5 knockdown (Fig. 4A). In addition, using the GEO dataset (GSE15499), the mRNA level of Maspin was found to be decreased by siHDAC5 in HUVEC (Fig. 4B). We designed an experiment to determine whether exosomal Maspin, which increases post-RT in a p53-dependent manner, is directly regulated by HDAC5. HepG2 cells were transfected with siHDAC5 and irradiated (4 Gy). Exosomal Maspin expression was verified by isolating the exosome released after 24 h. After control siRNA transfection, exosomal Maspin was increased by radiation. However, Maspin expression was not increased even after irradiation in exosomes from HDAC5-knock down HepG2 (Fig. 4C, D). Maspin has been studied to cause anti-angiogenic functions [18]. We verified the anti-angiogenic function of Maspin in human-derived vascular endothelial cells using HUVEC. An endothelial cell (EC) tube-forming assay was performed and the human recombinant Maspin was treated in a dose-dependent manner (Fig. 4E). Active EC branching and tubing were observed after VEGF treatment in HUVECs, whereas both branching and tubing were significantly reduced in a dose-dependent manner after treatment with human recombinant Maspin (Fig. 4F, G).

### Angiogenesis of HepG2-derived exosomes after RT

**Fig. 5:**
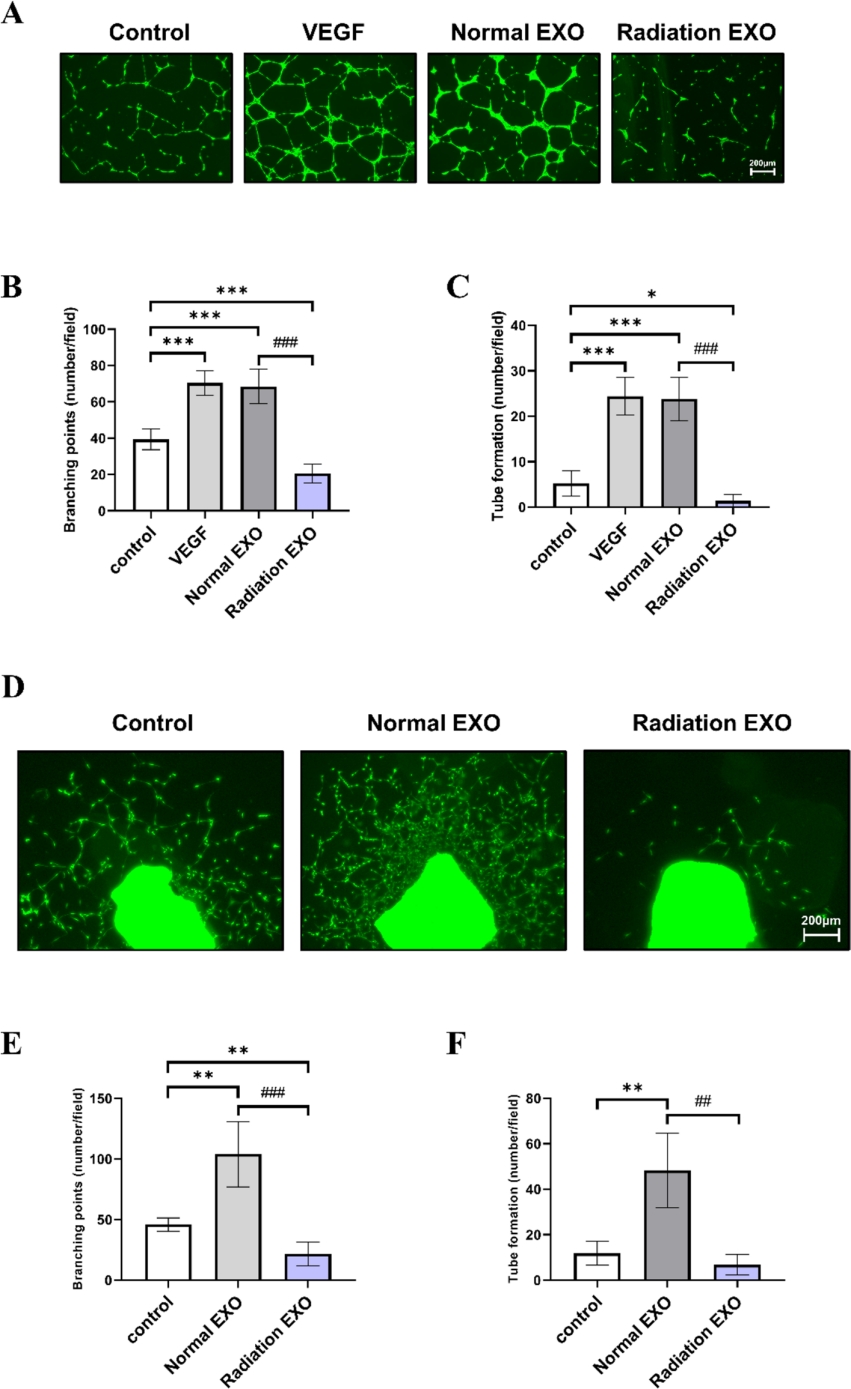
Inhibition of in vitro/ex vivo angiogenesis by treatment of RT-induced high Maspin exosomes. HUVECs were seeded on growth factor-reduced matrigel-coated plates and treated with VEGF (50 ng/mL), normal exosome (30 µg/mL), and radiation-derived exosome (30 µg/mL). After 16 h, calcein am dye (0.5 µg/mL) was added and fluorescence images were photographed using EVO M5000 Imaging System (Invitrogen) (**A**). Five locations of each well were randomly photographed, and the branching and tube organization were measured and compared with that of the control (**B**, **C**). Cell culture dishes were performed in at least three separate experiments. Scale bar = 200 μm. Data are shown as mean ± SEM. *P < 0.05, **P < 0.01, and ***P < 0.001 vs. control by Student’s t-test; # *P* < 0.05, ## *P* < 0.01, ### *P* < 0.001 vs. Normal exo by Student’s t-test. Aortas from 8-week-old C57BL/6 mice were isolated, cut into 1 mm sections, and then seeded on growth factor-reduced matrigel-coated plates. They were then treated with normal exosome (30 µg/mL) or radiation-derived exosome (30 µg/mL). After 5 days, they were stained with calcein am dye (0.5 µg/mL) and fluorescence images taken **(D).** Five aortic tissues of each well were randomly photographed, and the branching and tube organization were measured and compared with that of the control condition (**E**, **F**). Scale bar = 200 μm. *N* = 5 each, data are shown as mean ± SEM. *P < 0.05, **P < 0.01, ***P < 0.001 vs. Control by Student’s t-test; # *P* < 0.05, ## *P* < 0.01, ### *P* < 0.001 vs. Normal exo by Student’s t-test

Tumor-derived exosomes mainly function to increase tumor progression by changing the tumor microenvironment [19]. HepG2-derived exosomes are known to accelerate tumor progression by increasing angiogenesis [21, 22]. Maspin has anti-angiogenic functions [23]. We evaluated the anti-angiogenic response of exosomal Maspin by treating HUVECs with normal and radiation-derived exosomes secreted by HepG2. An endothelial cell (EC) tube-forming assay was performed, and HUVEC treated with normal-derived exosome showed active EC branching and tube-forming for angiogenesis. However, angiogenesis was significantly reduced in the radiation-derived exosome-treated group as compared with that in the control and normal-derived exosome group (Fig. 5A-C). To confirm a more direct effect at the vertebrate level, we performed the mouse aorta ring assay. As in the in vitro assay, a significantly increased aortic tissue-derived angiogenesis was observed in the normal-derived exosome group. Contrarily, angiogenesis was significantly reduced in the radiation-derived exosome-treated group (Fig. 5D-F).

### Alteration of exosomal miRNA expression by RT in HCC

**Fig. 6:**
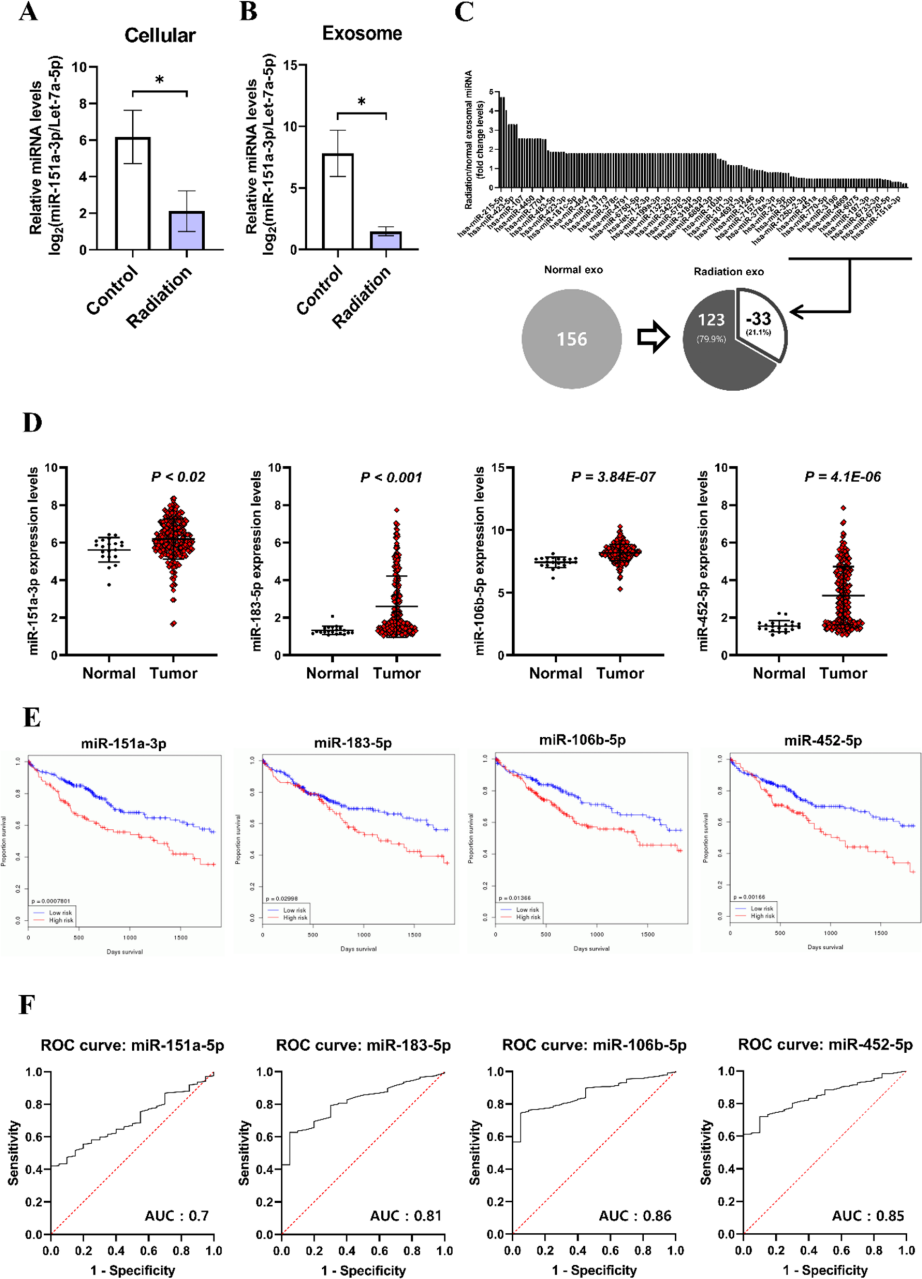
Expression analysis of exosomal miRNAs altered by RT in HCC and the clinical exploration and identification of candidates using bioinformatics analysis. The modulation of miR-151a-3p by radiation exposure was analyzed using qRT-PCR in the cell and exosome. (**A**, **B**). Data are shown as mean ± SEM. *P < 0.05 vs. Control by Student’s t-test. Before and after radiation exposure, exosomal miRNA was arranged into a fold change value and analyzed as a graph and ven diagram (**C**). Applying the GEO data set, the expression difference of each candidate miRNA between normal and cancer tissue was analyzed (**D**). To investigate the clinical significance of each candidate miRNA, OncomiR Cancer miRNome Atlas and patient survival were analyzed via Kaplan–Meier analysis (**E**). Based on the GEO data of four miRNAs in HCC tissue, the specificity and sensitivity are represented as the Area Under Curve (AUC) of the ROC curve (**F**)

Exosomal miRNAs substantially regulate tumor progression. miR-151a-3p is a miRNA closely related to p53 [24]. miR-151a-3p is loaded into the exosome and released, and miR-151a-3p-rich exosome derived from gastric cancer was found to promote liver metastasis [25]. We confirmed that p53 mRNA can be repressed through miR-151a-3p in miRNA target analysis (Fig. S10A). In addition, miR-151a-3p in cells and exosomes was significantly reduced by RT (Fig. 6A, B). Exosomal miRNA high-throughput sequencing was performed to detect variation in the miRNA configuration inside the exosome after RT. Exosomal miRNA variation altered by RT was clustered and confirmed using Heatmap (Fig. S7). To understand which intracellular function of the miRNAs inside the exosome changed after irradiation, it was classified and analyzed into various cell functional categories using Gene Ontology terms (Fig. S10B). Analyzing the fold change values of miRNAs, the graph was composed in descending order. RT significantly reduced thirty-three miRNAs among the 156 miRNAs inside the exosome (Fig. 6C). Among the miRNAs with reduced expression, those associated with cancer progression were analyzed using the GEO data set of HCC patients, which was downloaded from NCBI, and analyzed using volcano plot and mean-difference plot using the GEO2R analysis tool (Fig. S8 and 9). As a result of analysis utilizing the HCC miRNA GEO data set (Spain, GSE74618), four specific miRNAs (miR-151a-3p, miR-106b-5p, miR-183-5p, miR-452-5p) were found to be significantly increased in tumor tissues compared to normal levels (Fig. 6D) [20]. It was re-validated using another HCC miRNA GEO data set (Japan, GSE147889) to confirm the accuracy of the analysis results (Fig. S11). To ascertain the relationship between the four miRNAs with increased expression in HCC and the survival rate of HCC patients, we analyzed using OncomiR Cancer miRNome Atlas. High expression of four miRNAs in HCC patients was correlated significantly with poor survival (Fig. 6E) [26]. The AUC of the four miRNAs were as follows: miR-151a-3p AUC: 0.7, miR-106b-5p AUC: 0.86, miR-183-5p AUC: 0.81, and miR-452-5p AUC: 0.85 (Fig. 6F). We identified the molecular biological mechanisms of four miRNAs through KEGG pathway analysis (Table S1). We considered miR-151a-3p interesting and continued the study, and it was found to be related to the tumorigenesis of various cancers (Table S2). Furthermore, three cancer types were identified where patient survival was significantly associated with miR-151a-3p (Table S3). Collectively, RT can alter the composition of exosomal miRNAs released from HCC, which may contribute to tumor suppression. In particular, miR-151a-3p is associated with p53 and has a direct correlation with RT-induced exosomal component alteration in HCC.

### Regulation of p53-dependent radioresistance and epithelial-mesenchymal transition (EMT) by miR-151a-3p

**Fig. 7:**
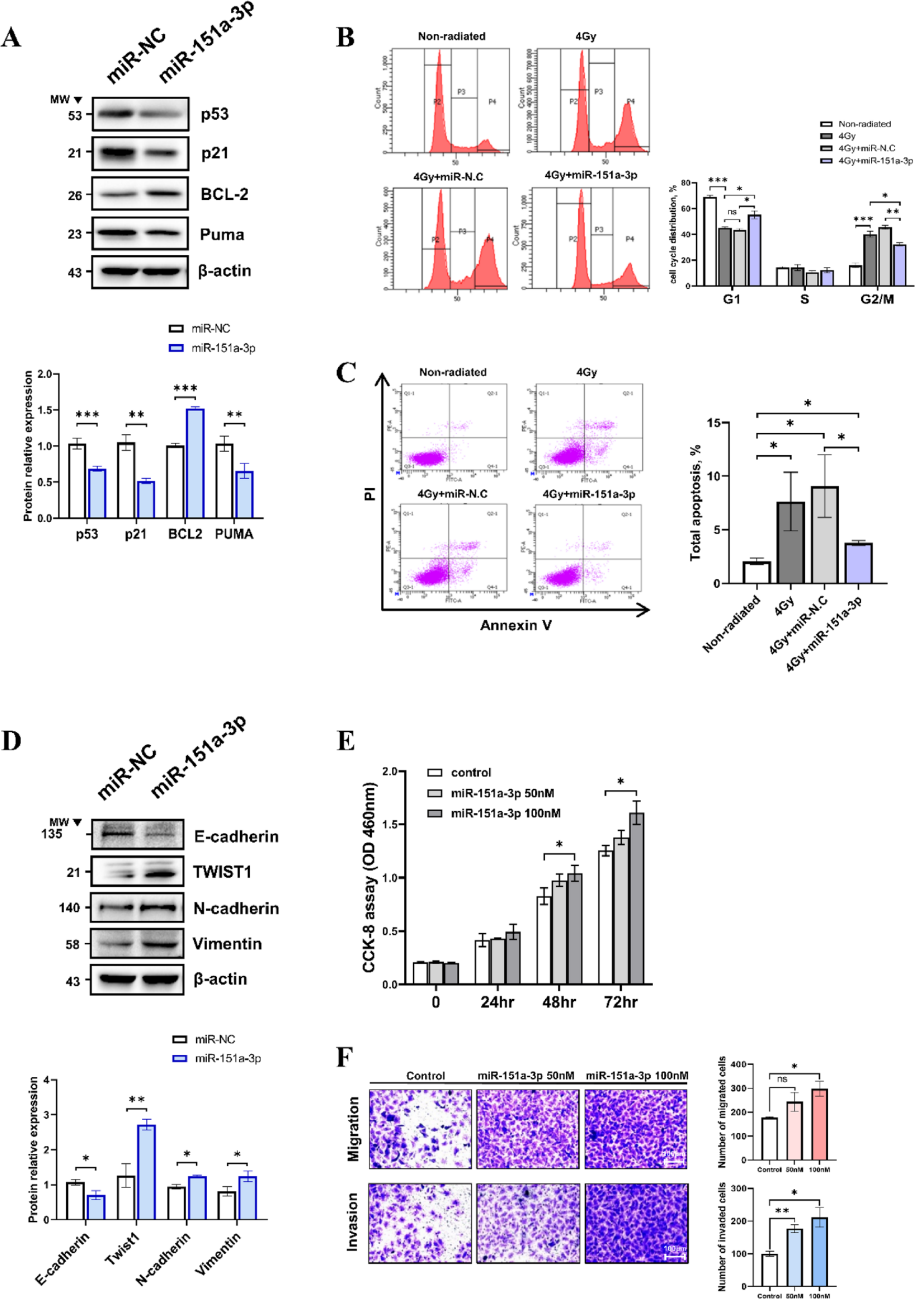
Radioresistance, proliferation, and EMT regulated by miR-151a-3p. Twenty-four hours after miR-151a-3p was transfected into HepG2, expression of p53 and p21, Puma, and Bcl-2 was confirmed by western blotting (**A**). Data are shown as mean ± SEM. *P < 0.05, **P < 0.01, and ***P < 0.001 vs. miR-NC by Student’s t-test. Cell cycle arrest assay using FACS was performed with PI staining to confirm that cell cycle arrest induced by radiation exposure was modulated by miR-151a-3p. Cell cycle was analyzed by dividing it into G1, S, and G2/M phases (**B**). Data are shown as mean ± SEM. *P < 0.05, **P < 0.01, and ***P < 0.001 vs. Non-radiated by Student’s t-test. To confirm that cell apoptosis induced by RT is modulated by miR-151a-3p, apoptosis was analyzed by Annexin V (FITC) and PI staining using FACS (**C**). Data are shown as mean ± SEM. *P < 0.05 vs. Non-radiated by Student’s t-test. Regulation of the representative epithelial-mesenchymal transition (EMT) markers E-cadherin, Twist1, Vimentin, and N-cadherin by miR-151a-3p was confirmed by western blot (**D**). Data are shown as mean ± SEM. *P < 0.05, **P < 0.01 vs. miR-NC by Student’s t-test. The cell proliferation according to the concentration of the miR-151a-3p was analyzed using CCK-8 assay (**E**). Data are shown as mean ± SEM. *P < 0.05 vs. Control by Student’s t-test. Cell migration and invasion ability according to the concentration of miR-151a-3p were analyzed using transwell assay. Five locations of each well were randomly photographed, and the migration and invasion were measured and compared with that of the control (**F**). Cell culture dishes were performed in at least three separate experiments. Scale bar, 100 µm. Data are shown as mean ± SEM. *P < 0.05, **P < 0.01 vs. control by Student’s t-test

Among the exosomal miRNAs strongly associated with poor survival of HCC, miR-151a-3p showed the most powerful tumor progression (fig S12-13). We investigated the radioresistance mechanism of miR-151a-3p. After overexpression was induced by transfection with miR-151a-3p mimic, the key molecules related to apoptosis and cell cycle were examined. p53 was considerably reduced, and the p53-dependent cell cycle and apoptosis molecules p21 and puma were correspondingly reduced. BCL2, a p53-dependent anti-apoptotic molecule that regulates the permeability of the mitochondrial outer membrane, was significantly increased. (Fig. 7A). Cell cycle analysis was conducted using FACS to verify the function of miR-151a-3p in cell cycle arrest induced by RT. The G2/M phase arrest was increased in the miR-N.C group and the 4 Gy-positive group. In the miR-151a-3p group, the G2/M phase arrest was reduced relatively (Fig. 7B). Additionally, miR-151a-3p inhibited cell apoptosis induced by RT. The miR-151a-3p group exhibited significantly reduced total cell apoptosis compared to miR-N.C and 4 Gy positive groups (Fig. 7C).

EMT, an important process in cancer metastasis, can be indirectly regulated by p53 [27, 28]. The expression of the EMT marker protein was verified 24 h after transfection of the negative control and miR-151a-3p in the cell by western blot. E-cadherin, a representative epithelial phenotype marker, was reduced in the miR-151a-3p group. The levels of mesenchymal phenotype marker vimentin and N-cadherin were inversely proportional to those of E-cadherin, and Twist1 expression was noticeably higher in association with p53 metastasis (Fig. 7D) [27, 29]. For functional evaluation that induces the progression of cancer of miR-151a-3p, proliferation, migration, and invasion assays were performed. A significant increase in proliferation was identified in the miR-151a-3p 100 nM group, depending on the concentration of miR-151a-3p (Fig. 7E). Similarly, tumor cell migration and invasion were remarkably increased in a concentration-dependent manner of miR-151a-3p (Fig. 7F). These studies clearly indicate that miR-151a-3p, whose exosome secretion is reduced by RT in HCC, increases radioresistance and can potentially induce tumor progression.

### Acceleration of tumor growth is attributable to miR-151a-3p overexpression

**Fig. 8:**
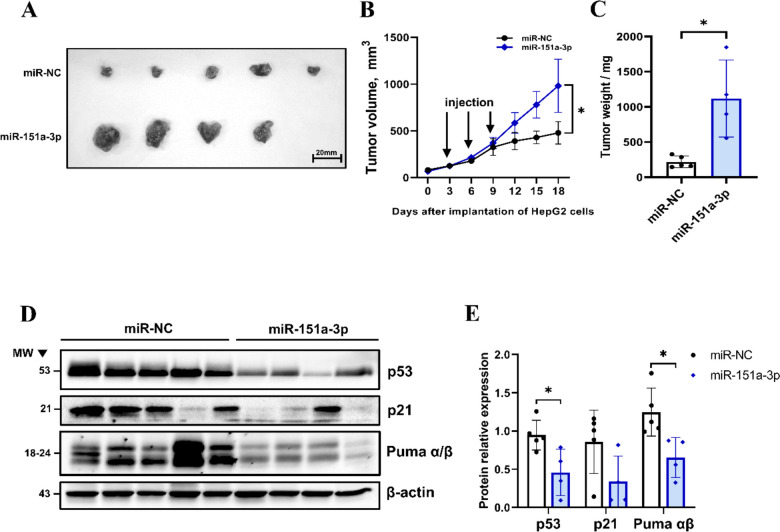
p53-suppressive tumor progression increased by miR-151a-3p overexpression in the HCC xenograft model. Acceleration of tumor growth by miR-151a-3p overexpression in a subcutaneous xenograft model (*N* = 5) (**A-C**). Representative images at day 18 post-transplantation of a subcutaneous xenograft nude mouse model injected with miR 151a-3p/miR-NC. Scale bar, 20 mm (**A**). In the HCC subcutaneous xenograft model, miR-151a-3p, miR-NC, and in vivo jet-PEI complex were injected thrice every three days from the third day after tumor transplantation, and the size of the tumors of each group was measured every three days (**B**). After the mice were sacrificed on the 18th day, the tumor was separated and the size was compared, and the weight of the tumors of each group was measured and plotted (**C**). The expression of the related target protein and downstream proteins in tumor tissues was analyzed by western blot (**D**, **E**). Data are shown as mean ± SEM. *P < 0.05 vs. miR-NC by Student’s t-test

To confirm the tumor progression capacity of miR-151a-3p in vivo, an HCC subcutaneous xenograft model was used. HepG2 cells (5 × 10^6^) were injected subcutaneously in the flank of nude mice. Thereafter, miR-NC and miR-151a-3p/in vivo jet-PEI complexes were injected thrice at 3-day intervals using the established in vivo jet-PEI transfection reagent to increase miR-151a-3p expression in cells [30]. The size of the tumor was significantly increased with miR-151a-3p overexpression, and the growth rate of the tumor was also faster (Fig. 8A-C). p53, P21, and Puma α/β expression was significantly inhibited in miR-151a-3p-overexpressing tumor tissues (Fig. 8D, E). These studies clearly indicate that overexpressed miR-151a-3p can significantly increase tumor growth and accelerate tumor progression by suppressing intratumoral p53 and its downstream signals.

### Anti-angiogenic and antitumor effect of RT-induced exosomes in HCC

**Fig. 9:**
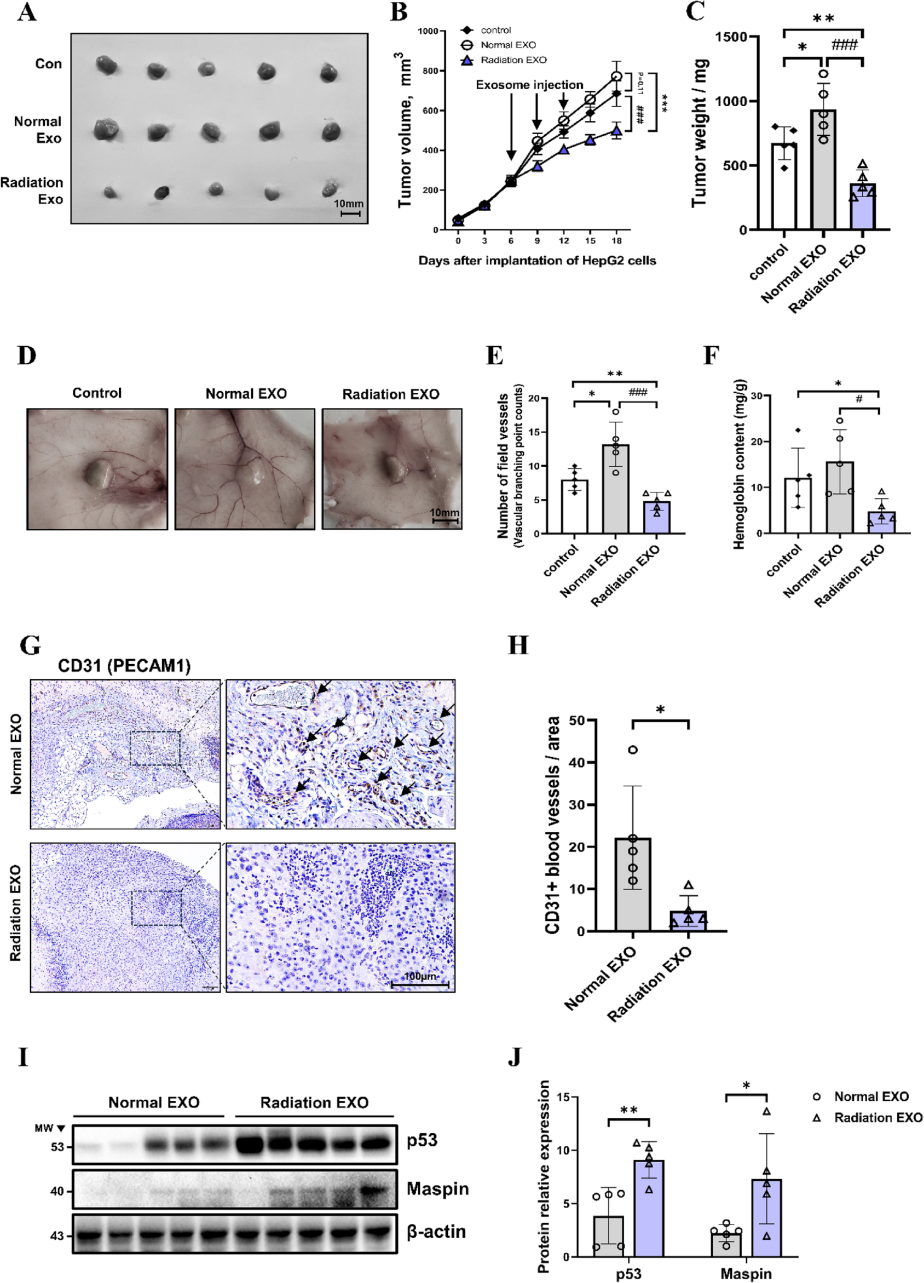
Inhibition of tumor angiogenesis and progression by RT-induced exosome in HCC xenograft model. Increased tumor growth in HCC xenograft model by normal- and RT-induced exosome injection (*N* = 5) (**A-C**). Representative images at day 18 post-transplantation of a subcutaneous xenograft nude mouse model injected with normal- and RT-induced exosomes Scale bar, 10 mm (**A**). In the HCC subcutaneous xenograft model, normal- and RT-induced exosomes were injected thrice every three days from the third day after tumor transplantation, and the size of the tumors of each group was measured every three days (**B**). After the mice were sacrificed on the 18th day, the tumor was separated and the weight of the tumors of each group was measured and plotted (**C**). data are shown as means ± SEM. *P < 0.05, **P < 0.01, and ***P < 0.001 vs. control by Student’s t test; #*P* < 0.05, ##*P* < 0.01, and ###*P* < 0.001 vs. normal EXO by Student’s t test. After the mice were sacrificed on the 18th day, tumors were isolated and angiogenesis around the tumors was photographed. Scale bar, 10 mm (**D**). The tumor vascular branching points of each mouse were counted and measured (**E**). Hemoglobin content (mg/g) was normalized by the weight of each tumor (**F**). data are shown as means ± SEM. *P < 0.05, **P < 0.01 vs. control by Student’s t test; #*P* < 0.05, ##*P* < 0.01, and ###P < 0.001 vs. normal EXO by Student’s t test. Xenograft tumor tissues were embedded in paraffin blocks, sectioned, and stained with hematoxylin and endothelium marker CD31 (PECAM1). CD31 + vessels were indicated by black arrows (**G**). Scale bar, 100 μm. CD31 + vessels within the tumors of each group were measured and quantified (**H**). The expression of p53 and Maspin in tumor tissue was expressed by Western blot and relative quantification was shown by graph (**I**, **J**). Data are shown as mean ± SEM. *P < 0.05, **P < 0.01 vs. Normal EXO by Student’s t-test

We have shown that the content of Maspin in RT-induced exosomes increased and miR-151a-3p decreased, thereby inhibiting angiogenesis and preventing tumor progression. Based on these results, an HCC subcutaneous xenograft model was used to verify whether normal and RT-induced exosome treatment resulted in angiogenesis and tumor growth inhibition in tumor progression. Normal exosome injection induced tumor growth, but RT-induced exosome showed rapid suppression of tumor growth after administration (Fig. 9A-C). The entire tumor vascular image was used to count the peritumoral angiogenesis in each group (Fig. 9D, E). Additionally, tumor tissue-derived hemoglobin content was measured to quantitatively evaluate intratumor angiogenesis (Fig. 9F). Compared to the control, the normal exosome injection group showed increased tumor-surrounding blood vessels, but RT-induced exosome resulted in significant suppression of peritumoral angiogenesis. We performed CD31 (PECAM1) immunostaining for histological analysis of blood vessels formed within the tumor (Fig. 9G). Compared to the normal exosome injection group, RT-induced exosome injection significantly suppressed the formation of CD31-positive vessels in tumor tissue (Fig. 9H). In contrast, RT-induced exosome injection remarkably increased the expression of p53 and Maspin in the tumor (Fig. 9I, J). Collectively, these in-vivo studies suggest that altered exosomal components by RT resulted in tumor angiogenesis and growth inhibition in HCC. These studies can be an effective strategy to support radiotherapy.

## Discussion

Exosomes, which are widely present in various body fluids, contain various proteins, miRNAs, and small molecules [31]. After secretion, exosomes are absorbed by adjacent or distant cells, and the protein and miRNAs in them can promote tumor growth, invasion, angiogenesis, and resistance to RT. Therefore, exosomal miRNAs are promising as biomarkers in clinical targeted therapies that regulate cancer progression [32]. RT is an important treatment for preventing tumor progression, and through various mechanisms, it affects target cancer tissue and surrounding cancer cells. Among these mechanisms, p53 plays a key role in suppressing cancer through RT [33, 34].

Here, we demonstrate a previously unknown interaction of p53 and HDAC5 in HCC by RT. Moreover, the expression of both proteins was confirmed in a time-dependent manner by RT (Fig. 1). We discovered that the expression of HDAC5 was restored to its original state 24 h after RT of 2 Gy. Similarly, p53 has been shown to exhibit expression dynamics Responses after irradiation and it determined cell fate [35]. In Fig. 2E, it was verified that p53 expression was regulated by siHDAC5 transfection. We consider that the dynamics of HDAC5 by RT may contribute to determining cell fate through regulating p53 expression. Therefore, it will be covered in further studies.

HDAC5 expression was increased by RT in p53-/-HepG2 cells, indicating that HDAC5 upregulates p53 (Fig. 2). Therefore, HDAC5 plays an important role as an upstream regulator of p53-mediated exosome secretion by RT. In fact, in the mRNA seq data of cancer tissues and paired adjacent tissues of HCC patients, the expression of HDAC5 was significantly increased in cancer tissues (Fig. S5). This suggests that cancer itself can upregulate HDAC5 for hypersecretion of exosomes to regulate the intra-tumoral microenvironment.

TSAP6 is known to tightly regulate exosome secretion [10]. TSAP6 as exosome secretion-modulating protein and regulated through p53, showed increased expression upon RT. The presence or absence of the p53 gene dramatically changed exosome secretion depending on the time of RT. TSAP6 in p53 + / + cells had the highest expression after 12 h of RT, and secretion of exosome was also highest after 12 h of RT. Contrarily, TSAP6 expression after RT in the p53-/- cells was unchanged, and exosome secretion after RT decreased (Fig. 2D & 3). In addition, TSAP6 was elaborately regulated by HDAC5-mediated p53. HDAC5 upregulated by RT increased the expression of both p53 and TSAP6, but HDAC5 knockdown inhibited their upregulation by RT (Fig. 2E). Therefore, TSAP6 is clearly involved in exosome secretion and is regulated by HDAC5-mediated p53 resulting from RT.

Among the exosomal proteins, Maspin is regulated by the p53 gene. Inside the exosome, Maspin is increased by radiation exposure in the lung cancer cell line [13]. We found that the RT-induced release of exosomal Maspin was regulated in HCC in a p53-dependent manner (Fig. 3B). Interestingly, it was discovered that the RT-induced release of exosomal Maspin was regulated by HDAC5 (Fig. 4A-D). Maspin is loaded on the exosome and released outside the cell. It suppresses angiogenesis and tumor progression in the tumor microenvironment. We confirmed that angiogenesis was significantly reduced after treating HUVECs with radiation-induced high Maspin expressing exosomes. In the angiogenesis assay using mouse aortic vascular tissue, angiogenesis was especially reduced in the radiation exosome-treated group compared with that in normal exosome-treated group (Fig. 5). Angiogenesis, which is essential for tumor growth and metastasis, was significantly inhibited by exosomal Maspin increased by RT, and this can be considered a benefit of RT. Moreover, RT-induced cellular and exosomal Maspins in HCC are tightly regulated by HDAC5 (Fig. 4A-D).

The exosome is loaded with various molecules (protein, mRNA, miRNA, etc.) and plays an important role in communication between cells. Among the loaded molecules, miRNA is a potential biomarker and is under extensive study [36, 37]. After RT, changes in the miRNA composition balance inside the exosomes were observed. The change in miRNA inside exosomes due to RT was thought to be the starting point for conversions in the tumor microenvironment.

We confirmed that miR-151a-3p was regulated by RT at the cellular and exosomal levels and that it directly inhibited p53 (Fig. 6A, B & Fig. 7A). Therefore, we performed an exosomal miRNA high-throughput sequencing to analyze the overall miRNA expression in exosomes released in culture and exosomes released from irradiated cells. As per the result of the analysis, 156 exosomal miRNAs out of a total of 2588 miRNAs were expressed. Among them, we analyzed 33 miRNAs whose expression almost disappeared after RT (Fig. 6C). To identify the relationship between the disappeared 33 miRNAs and HCC, each miRNA expression was analyzed using the miRNA microarray GEO dataset cohort of HCC patients. Among the 33 miRNAs that disappeared after RT, miR-151a-3p, miR-106b-5p, miR183-5p, and miR452-5p involved in cell proliferation and invasion were significantly highly expressed in HCC (Fig. 6D) [25, 38–40].

Subsequently, we used the TCGA database (oncomiR) to investigate the association between four miRNAs and HCC patient survival rates. All miRNAs were directly correlated with the poor prognosis of HCC patients (Fig. 6E). In the specificity and sensitivity analysis using the AUROC curve, all four miRNAs showed significantly high AUC levels (Fig. 6F). These results are expected to contribute to the mechanism of RT for inhibiting cancer growth.

P53 as a transcription factor was significantly suppressed by miR-151a-3p, and its downstream signaling was also extensively affected. Also, by inhibiting the expression of p53 downstream molecules p21 and Puma, which play an important role in the cell cycle and apoptosis in the p53 signaling pathway, miR-151a-3p prevented cell cycle arrest and suppressed apoptosis after RT. Eventually, we confirmed that miR-151a-3p induces radiation resistance by inhibiting p53 signaling (Fig. 7A-C).

Loss of p53 function can influence cancer metastasis [27]. We confirmed that EMT was significantly advanced by miR-151a-3p-induced inhibition of p53. In particular, the expression of Twist1, Vimentin, and N-cadherin, which can be directly inhibited by p53, was increased by p53 inhibition via miR-151a-3p (Fig. 7D-F). An increase in Twist1 can indirectly inhibit cell cycle arrest and apoptosis and promote cancer metastasis [29, 41]. Finally, we confirmed the acceleration of cancer growth by miR-151a-3p overexpression using an HCC xenograft model. miR-151a-3p significantly suppressed p53 and downstream signals in tumor tissues, which rapidly promoted cancer growth compared to the control (Fig. 8). Furthermore, RT-induced exosome treatment showed more powerful inhibition of peritumoral angiogenesis and tumor growth in the HCC xenograft model. In particular, compared to normal exosome administration, RT-induced exosome injection showed significantly more intratumoral angiogenesis and caused increased p53 expression due to exosomal miR-151a-3p regulation. The expression of Maspin, which inhibits angiogenesis, was also increased through intratumor injection of RT-induced exosomes (Fig. 9).

## Conclusions

**Fig. 10:**
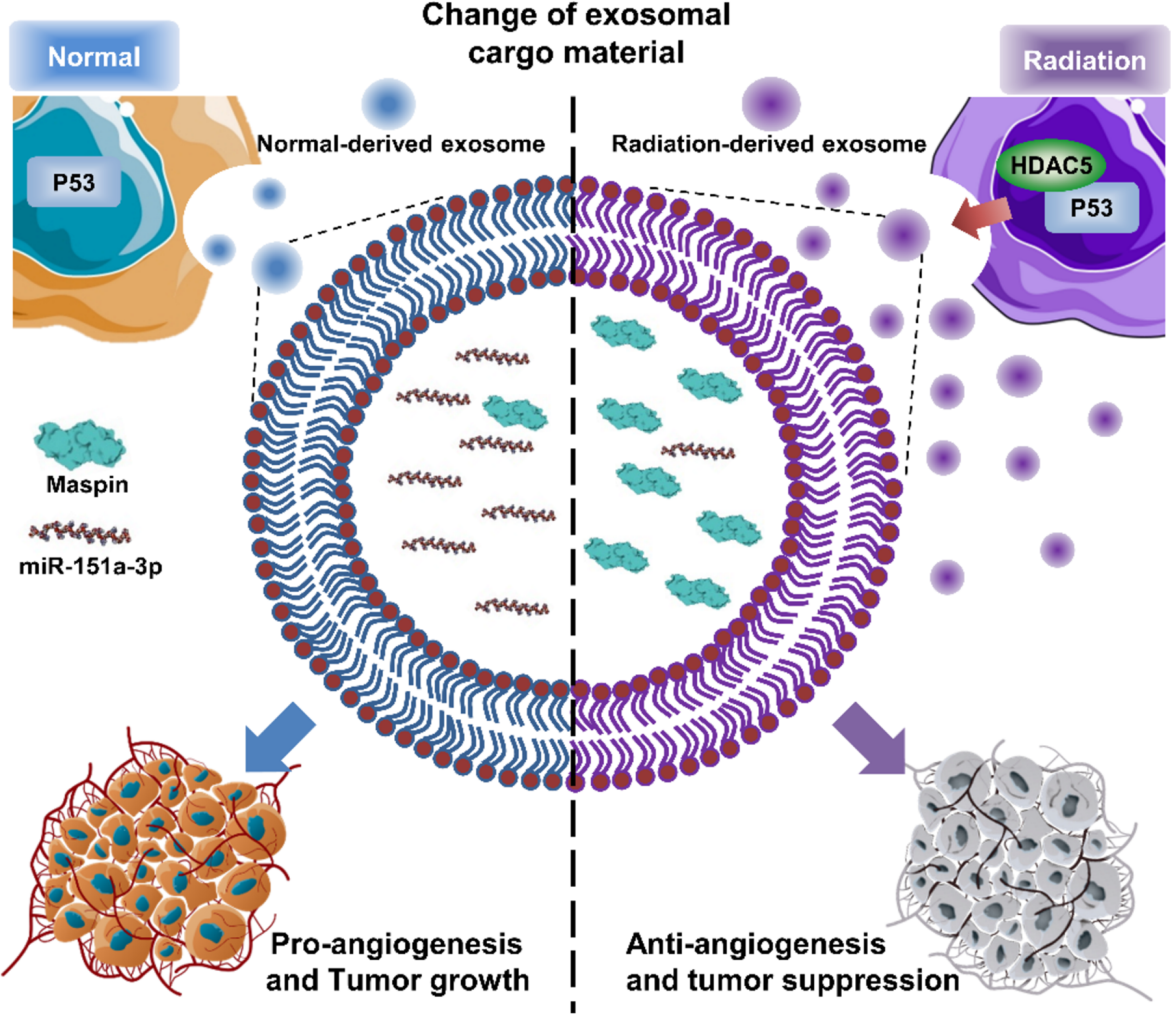
Exosomal Maspin and miR-151a-3p: Biomarkers Enhancing Radiation Treatment Sensitivity in HCC. A schematic image; In HCC, RT can alter exosomal components by mediating HDAC5 and p53, and exosomal Maspin and miR-151a-3p have the potential as biomarkers that can modulate radiosensitivity

Collectively, we studied the sophisticated regulation of the exosomal component caused by RT-induced HDAC5-p53 interaction in HCC. HDAC5 and p53 interacted upon exposure to RT, which increased exosome release and altered the exosomal components. HDAC5 was identified as an upstream regulator of Maspin, and HDAC5 overexpression induced by RT increased cellular and exosomal Maspin expression. Treatment with RT-induced high Maspin expressing exosomes significantly inhibited angiogenesis in HUVECs. In addition, exosomal miR-151a-3p, an miRNA capable of suppressing p53 to promote HCC, was significantly reduced at both cellular and exosomal levels after RT. Using exosomal miRNA sequencing, we found miRNAs that were inversely associated with the survival rate of HCC patients. They showed significant AUC levels that can diagnose HCC. Among them, exosomal miR-151a-3p inhibited p53 in HCC, induced radioresistance and EMT, and accelerated tumor growth. Finally, compared to normal exosomes, RT-induced exosomes showed powerful intratumoral angiogenesis and tumor growth inhibitory effects (Fig. 10).

In conclusion, our findings establish HDAC5 as a critical component determining the p53-mediated release of exosomes and alteration of exosomal components by RT in HCC. We especially identified that regulation of exosomal Maspin and miR-151a-3p improve sensitivity to RT. Understanding the molecular mechanisms of exosome secretion and exosomal components will help design novel therapeutics that improve sensitivity to RT in HCC.

### Supplementary Information

**Additional file 1: Supplementary figure 1.** Using CRISPR-Cas9 system, a cell line with p53 gene deleted was manufactured in HepG2. We deleted the p53 gene in the p53+/+ HepG2 cell line using the CRISPR-Cas9 system. The target site of Human p53: TGTAACAGTTCCTGCATGGG was investigated by the NGS method. The insertion/deletion frequency was 99.98% (T insertion:19.9%, CCTG deletion:18.8%, AT insertion:19.5%).** Supplementary figure 2.** Measurement and identification of differences in cell cycle arrest effects according to the presence of the p53 gene in HCC by RT. Changes in cell cycle after irradiation using HepG2, a liver cancer cell line, were measured by FACS. This was done to confirm the radiation resistance effect of conventional p53. Using FACS equipment, it was confirmed that cell cycle arrest was induced in p53+/+, and p53-/-cells by radiation treatment. In p53+/+, powerful cell cycle arrest in the G2/M phase was observed from 6 hours after irradiation and gradually switched to G1 phase cell cycle arrest as time passed. A similar cell cycle alteration was observed in p53-/-cell, but it was verified that the effect was smaller than that of p53+/+cell.** Supplementary figure 3.** The importance of the P53 gene in RT-induced apoptosis. Changes in apoptosis after irradiation using HepG2, a liver cancer cell line, were measured by FACS. Using FACS equipment, it was confirmed that apoptosis is induced in p53+/+, p53-/-cells by radiation treatment. p53-/-cell had significantly less apoptosis 48 hours after irradiation than p53+/+cell. It showed stronger cell cycle arrest and apoptosis at p53+/+cell compared to p53-/-cell.** Supplementary figure 4.** RT-induced cellular localization of HDAC5 and p53. To examine shifts in HDAC5 and p53 localization caused by RT, HepG2 was infected with GFP-HDAC5 adenovirus. After exposure to 4 Gy of radiation, time-dependent localization of HDAC5 and p53 was confirmed by immunofluorescence.** Supplementary figure 5.** Differences in HDAC5 expression in tumor and surrounding tissues in HCC. Using the GEO dataset (GSE84005 and GSE104310), HDAC5 expression was analyzed in HCC patient tumor tissues and paired adjacent tissues, respectively. Data are shown as mean±SEM. *P < 0.05, **P < 0.01, and ***P < 0.001 vs. paired adjacent tissues by Student’s t-test.** Supplementary figure 6.** Measurement of exosome secretion induced by irradiation. To quantitatively and qualitatively measure the amount of exosome released from HepG2 cells by irradiation, it was measured using the NANOSIGHT instrument using the Nanoparticle Tracking Analysis technique (A, B).** Supplementary figure 7.** Measurement and verification of changes in microRNA composition inside the exosome due to irradiation. Exosomal miRNAs changed after radiation exposure was analyzed through heatmap using the Mev software (Small RNA sequencing, miRNA-Seq Only Analysis Program developed by Dana-Farber Cancer Institute in the United States).** Supplementary figure 8.** Bioinformatics analysis to explore for candidate groups in databases of previous studies. We analyzed the search for candidates with significant values using volcanic plots and mean-difference plots to investigate promising candidates from this study data and two previous study databases. Data with significant *P*-value and expression level were displayed in red for positive values and blue for negative values (GSE74618).** Supplementary figure 9.** Secondary analysis of candidates using the HCC patient database of previous studies. We analyzed the search for candidates with significant values using volcanic plots and mean-difference plots to investigate promising candidates from this study data and two previous study databases. Data with significant *P*-value and expression level were displayed in red for positive values and blue for negative values (GSE147889).** Supplementary figure 10.** Protein target analysis and intracellular function analysis using exosomal miRNA candidates. Targetscan (http://www.targetscan.org/vert_72/) analysis tool was used to analyze the target protein of each miRNA. As a result of the target analysis of miRNA, it was discovered that miRNA151a-3p can bind to the p53 mRNA target region with a high probability (A). The Gene Ontology term was used to discriminate the cellular functions of control and radiation-derived exosomal miRNAs (B).** Supplementary figure 11.** Expression differences in HCC adjacent tissues and tumor tissues of exosomal miRNAs reduced by RT. Using the GEO dataset (GSE147889), differences in the expression of four exosomal miRNA candidates were investigated in adjacent tissues and tumor tissues of HCC patients. tumor tissues vs. adjacent tissues by Student’s t-test.** Supplementary figure 12.** HCC proliferation assay of reduced miR-106b-5p, miR-183-5p, and miR-452-5p in exosomes after RT. HepG2 was transfected with 100 nM of miR-106b-5p, miR-183-5p, and miR-452-5p, respectively, and cell proliferation was investigated at 24-hour intervals up to 72 hours using the CCK-8 assay.** Supplementary figure 13.** Cell migration assay of reduced miR-151a-3p, miR-106b-5p, miR-183-5p, and miR-452-5p in exosomes after RT. HepG2 was transfected with 100 nM each of miR-151a-3p, miR-106b-5p, miR-183-5p, and miR-452-5p, and a wound healing assay was performed to verify cell migration. Cell culture dishes were performed in at least three separate experiments. Data are shown as mean±SEM. *P < 0.05 vs. control by Student’s t-test.

## Data Availability

Not applicable.

## Abbreviations

RT: Radiation treatment
HDAC5: Histone deacetylase 5
HCC: Hepatocellular carcinoma
TSAP6: Tumor Suppressor Activation Pathway 6
miRNA: MicroRNA
EMT: Epithelial-mesenchymal transition
