# Supplementary material for: HDAC5-mediated exosomal Maspin and miR-151a-3p as biomarkers for enhancing radiation treatment sensitivity in hepatocellular carcinoma
Source: Biomater Res. 2023 Dec 15;27:134. doi: 10.1186/s40824-023-00467-7 (PMC10725039; doi:10.1186/s40824-023-00467-7)

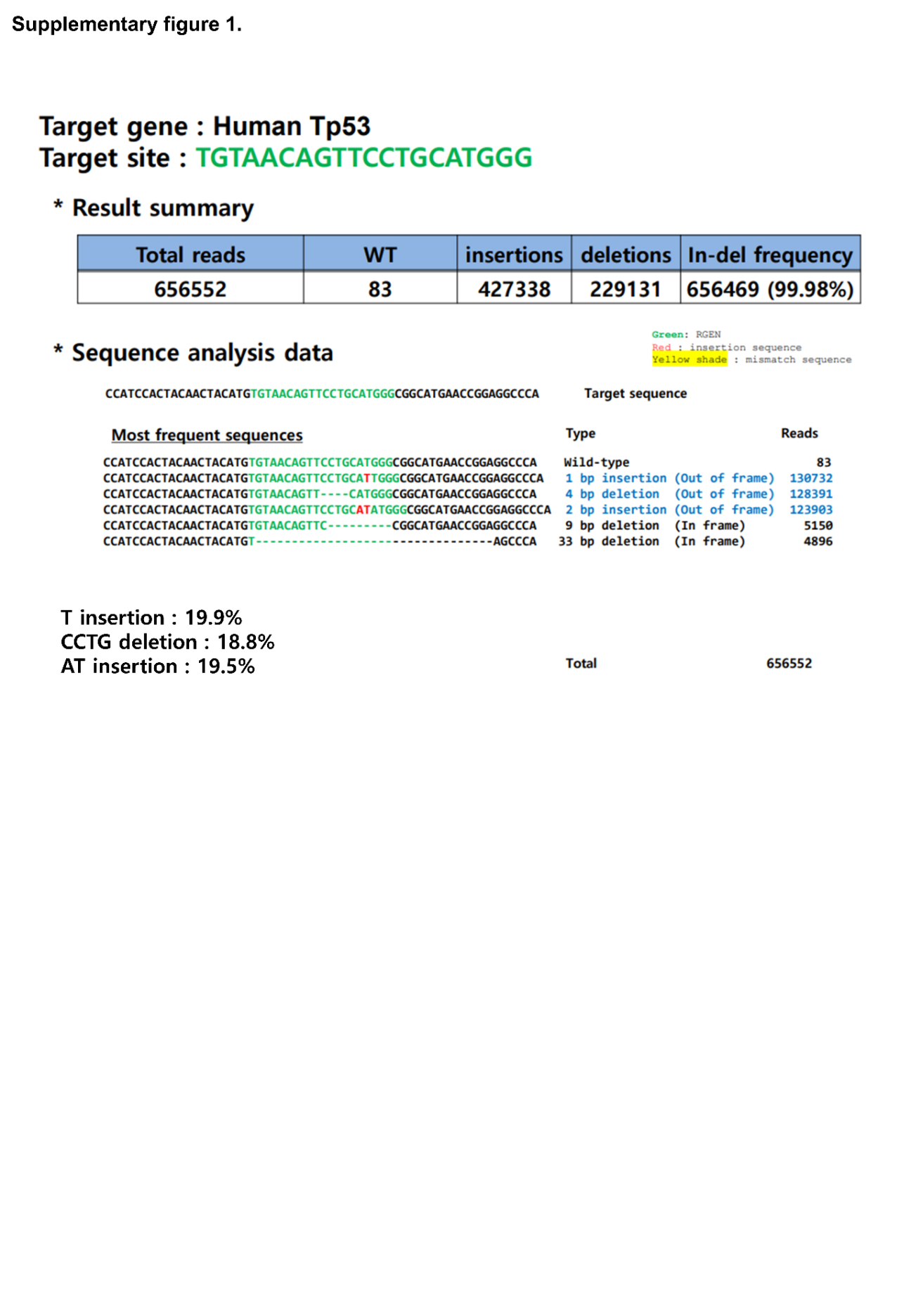


**Supplementary figure 1. Using CRISPR-Cas9 system, a cell line with p53 gene deleted was manufactured in HepG2**

We deleted the p53 gene in the p53+/+ HepG2 cell line using the CRISPR-Cas9 system. The target site of Human p53: TGTAACAGTTCCTGCATGGG was investigated by the NGS method. The insertion/deletion frequency was 99.98% (T insertion:19.9%, CCTG deletion:18.8%, AT insertion:19.5%).


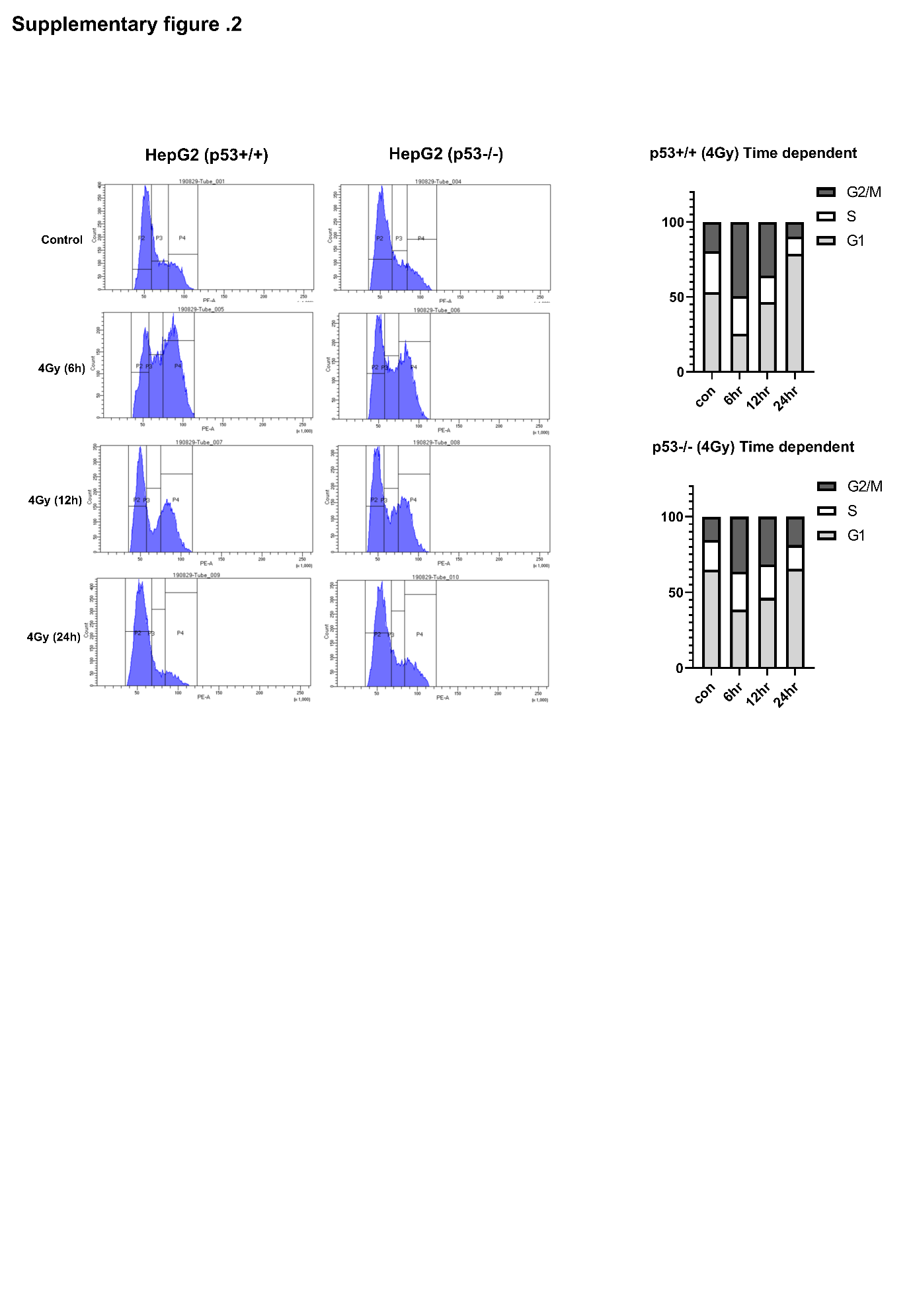


**Supplementary figure 2. Measurement and identification of differences in cell cycle arrest effects according to the presence of the p53 gene in HCC by RT**

Changes in cell cycle after irradiation using HepG2, a liver cancer cell line, were measured by FACS. This was done to confirm the radiation resistance effect of conventional p53. Using FACS equipment, it was confirmed that cell cycle arrest was induced in p53+/+, and p53-/-cells by radiation treatment. In p53+/+, powerful cell cycle arrest in the G2/M phase was observed from 6 hours after irradiation and gradually switched to G1 phase cell cycle arrest as time passed. A similar cell cycle alteration was observed in p53-/-cell, but it was verified that the effect was smaller than that of p53+/+cell.


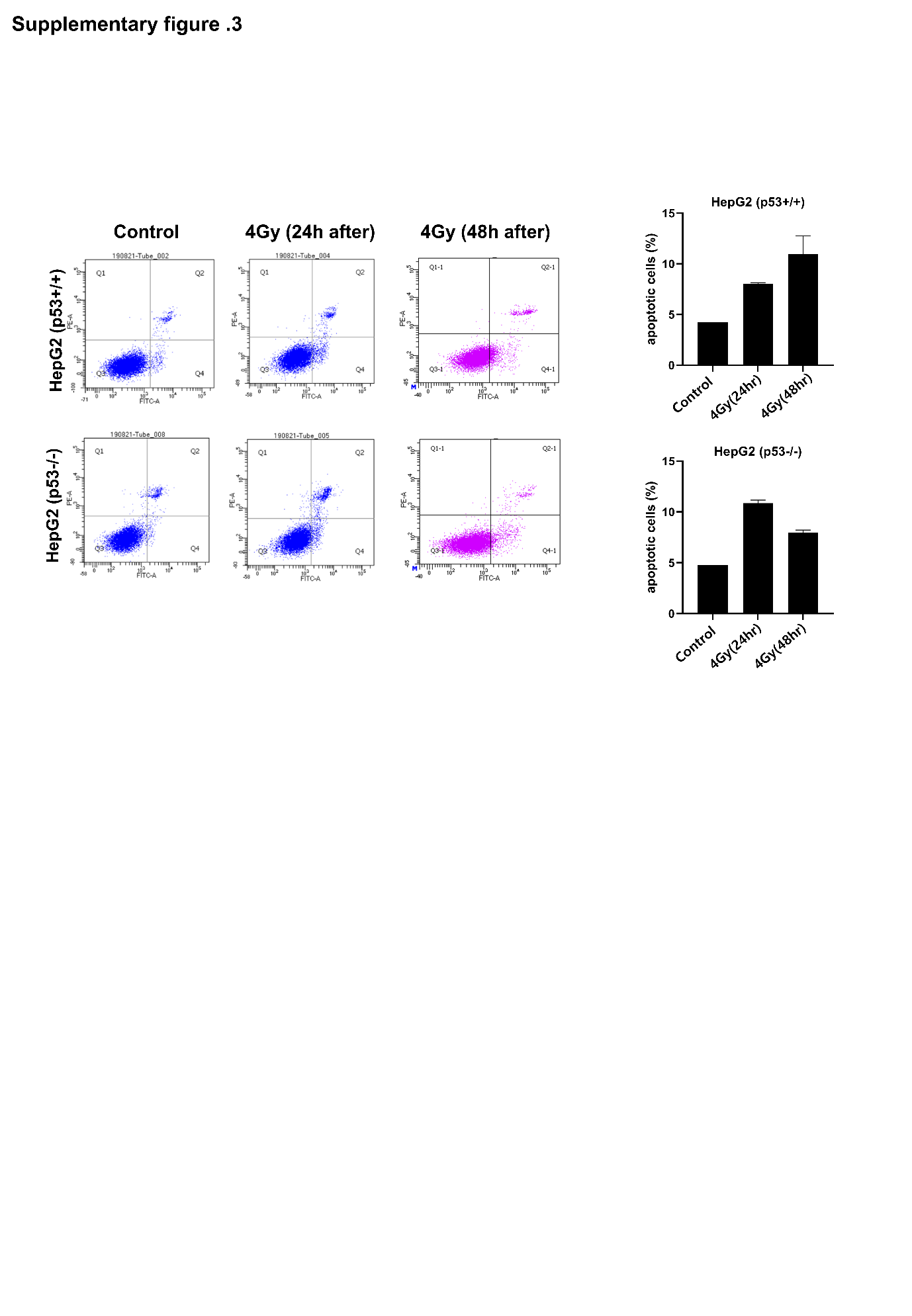


**Supplementary figure 3. The importance of the P53 gene in RT-induced apoptosis**

Changes in apoptosis after irradiation using HepG2, a liver cancer cell line, were measured by FACS. Using FACS equipment, it was confirmed that apoptosis is induced in p53+/+, p53-/-cells by radiation treatment. p53-/-cell had significantly less apoptosis 48 hours after irradiation than p53+/+cell. It showed stronger cell cycle arrest and apoptosis at p53+/+cell compared to p53-/-cell.


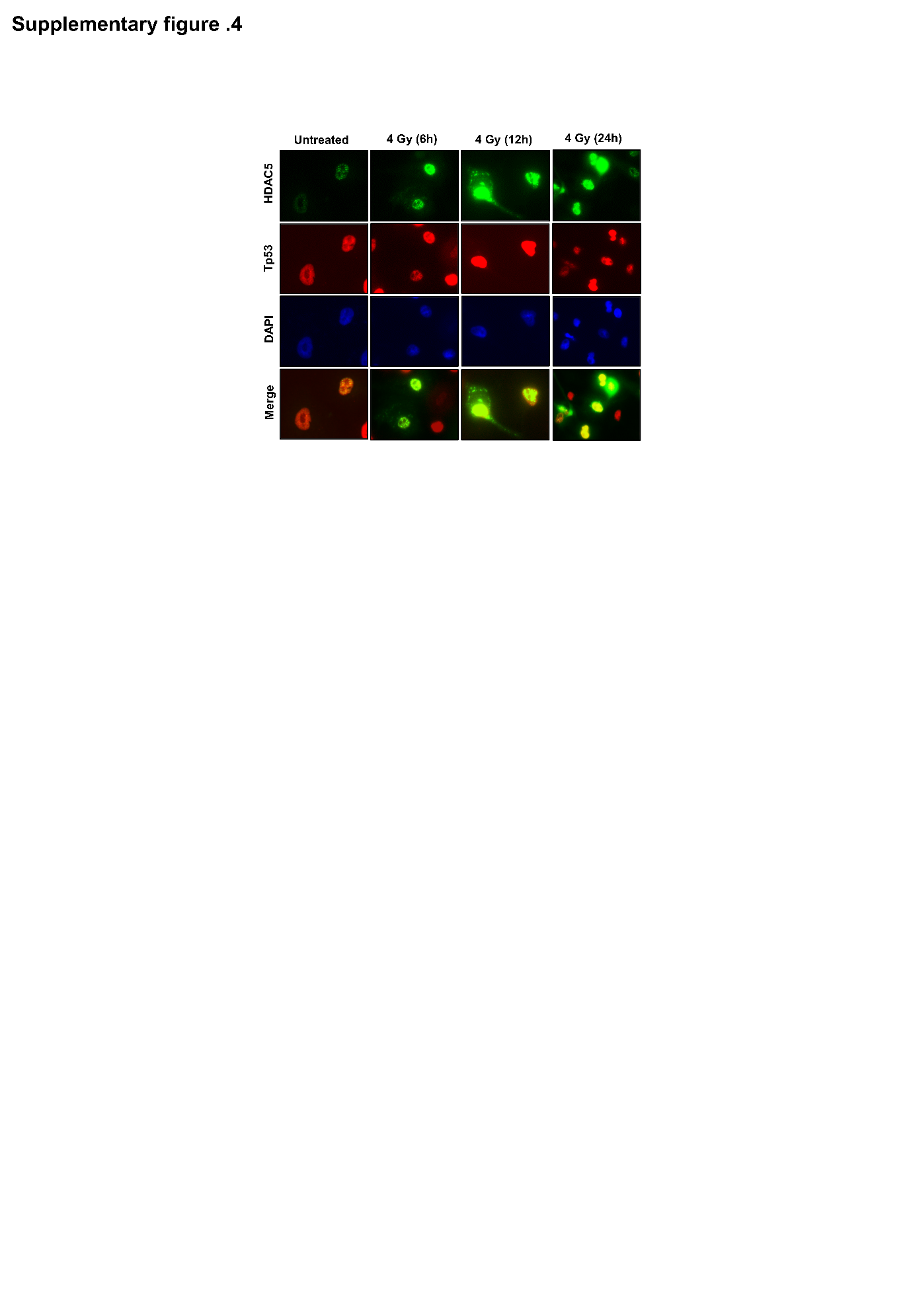


**Supplementary figure 4. RT-induced cellular localization of HDAC5 and p53**

To examine shifts in HDAC5 and p53 localization caused by RT, HepG2 was infected with GFP-HDAC5 adenovirus. After exposure to 4 Gy of radiation, time-dependent localization of HDAC5 and p53 was confirmed by immunofluorescence.


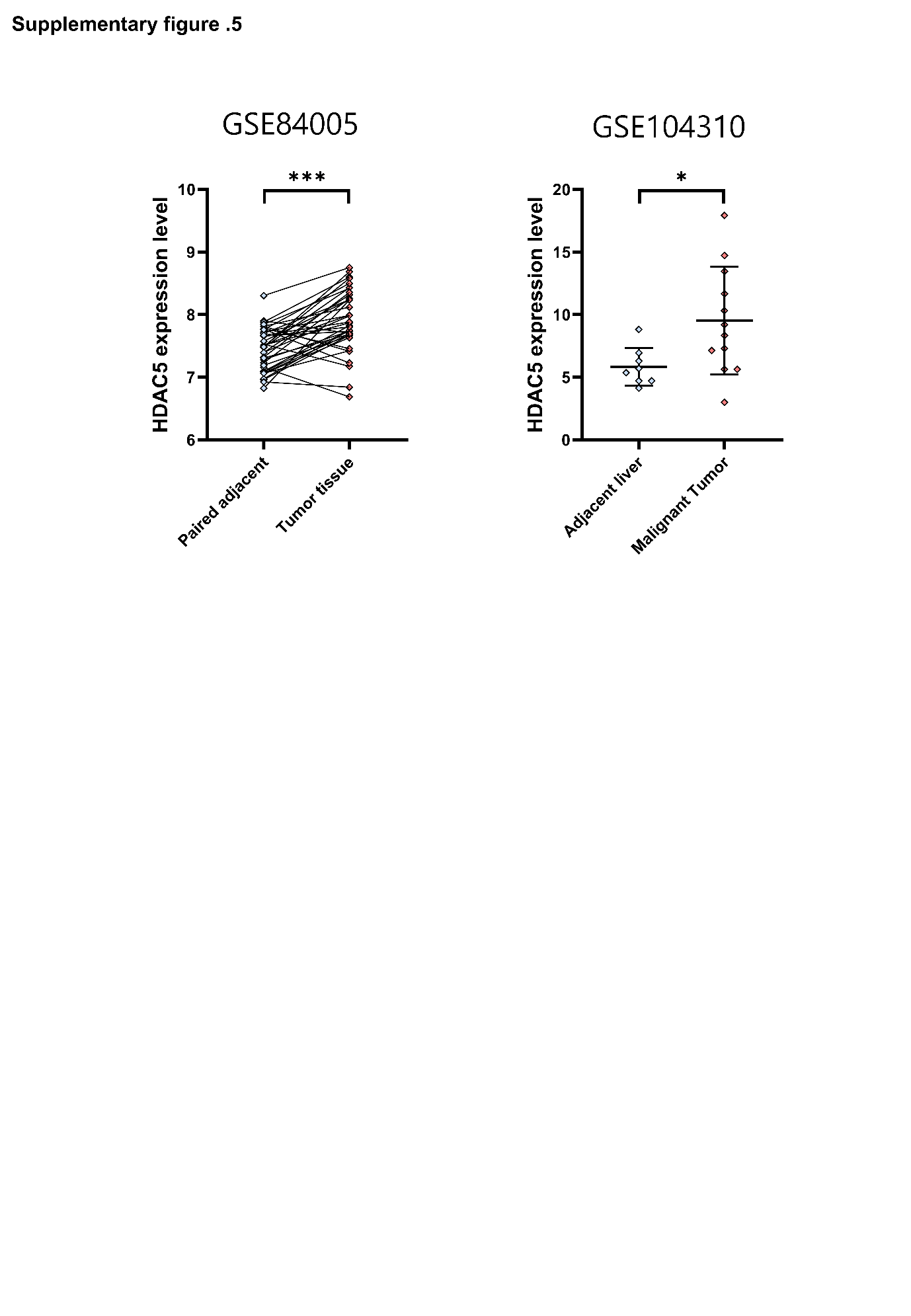


**Supplementary figure 5. Differences in HDAC5 expression in tumor and surrounding tissues in HCC**

Using the GEO dataset (GSE84005 and GSE104310), HDAC5 expression was analyzed in HCC patient tumor tissues and paired adjacent tissues, respectively. Data are shown as mean±SEM. *P < 0.05, **P < 0.01, and ***P < 0.001 vs. paired adjacent tissues by Student’s t-test.

**
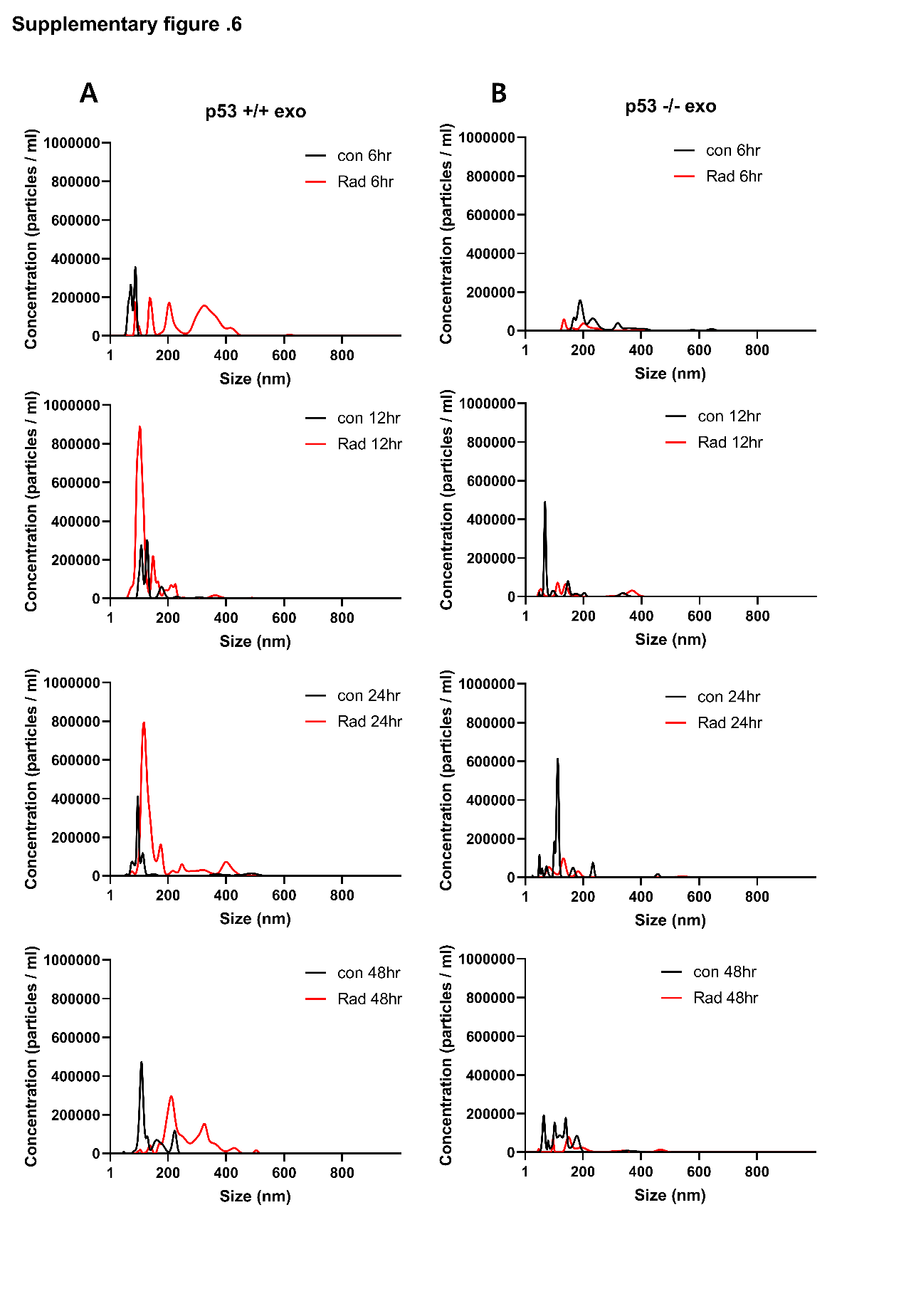
Supplementary figure 6. Measurement of exosome secretion induced by irradiation.**

To quantitatively and qualitatively measure the amount of exosome released from HepG2 cells by irradiation, it was measured using the NANOSIGHT instrument using the Nanoparticle Tracking Analysis technique **(A,B).**


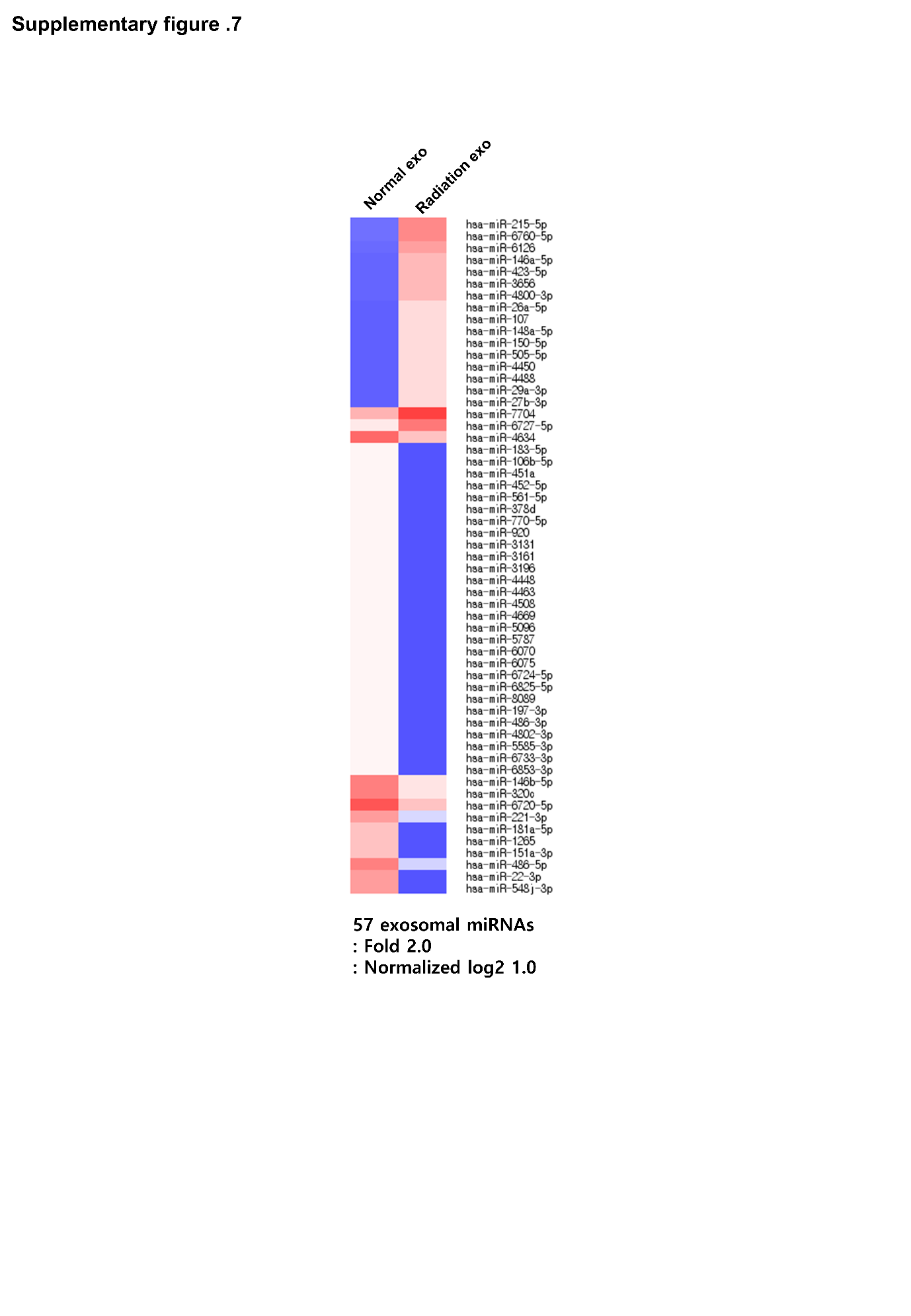


**Supplementary figure 7. Measurement and verification of changes in microRNA composition inside the exosome due to irradiation**

Exosomal miRNAs changed after radiation exposure was analyzed through heatmap using the Mev software (Small RNA sequencing, miRNA-Seq Only Analysis Program developed by Dana-Farber Cancer Institute in the United States).


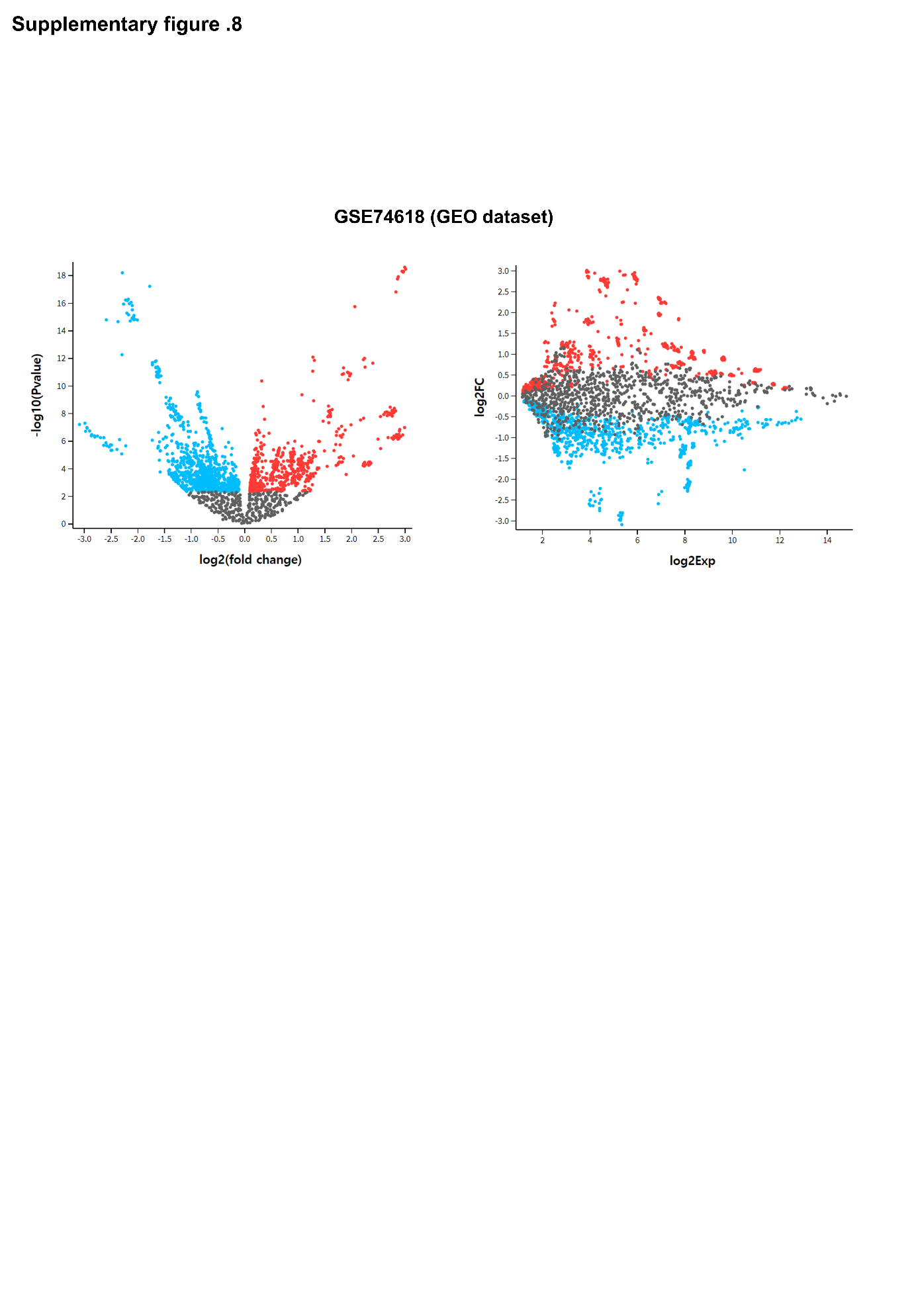
**Supplementary figure 8. Bioinformatics analysis to explore for candidate groups in databases of previous studies**

We analyzed the search for candidates with significant values using volcanic plots and mean-difference plots to investigate promising candidates from this study data and two previous study databases. Data with significant P-value and expression level were displayed in red for positive values and blue for negative values (**GSE74618**).


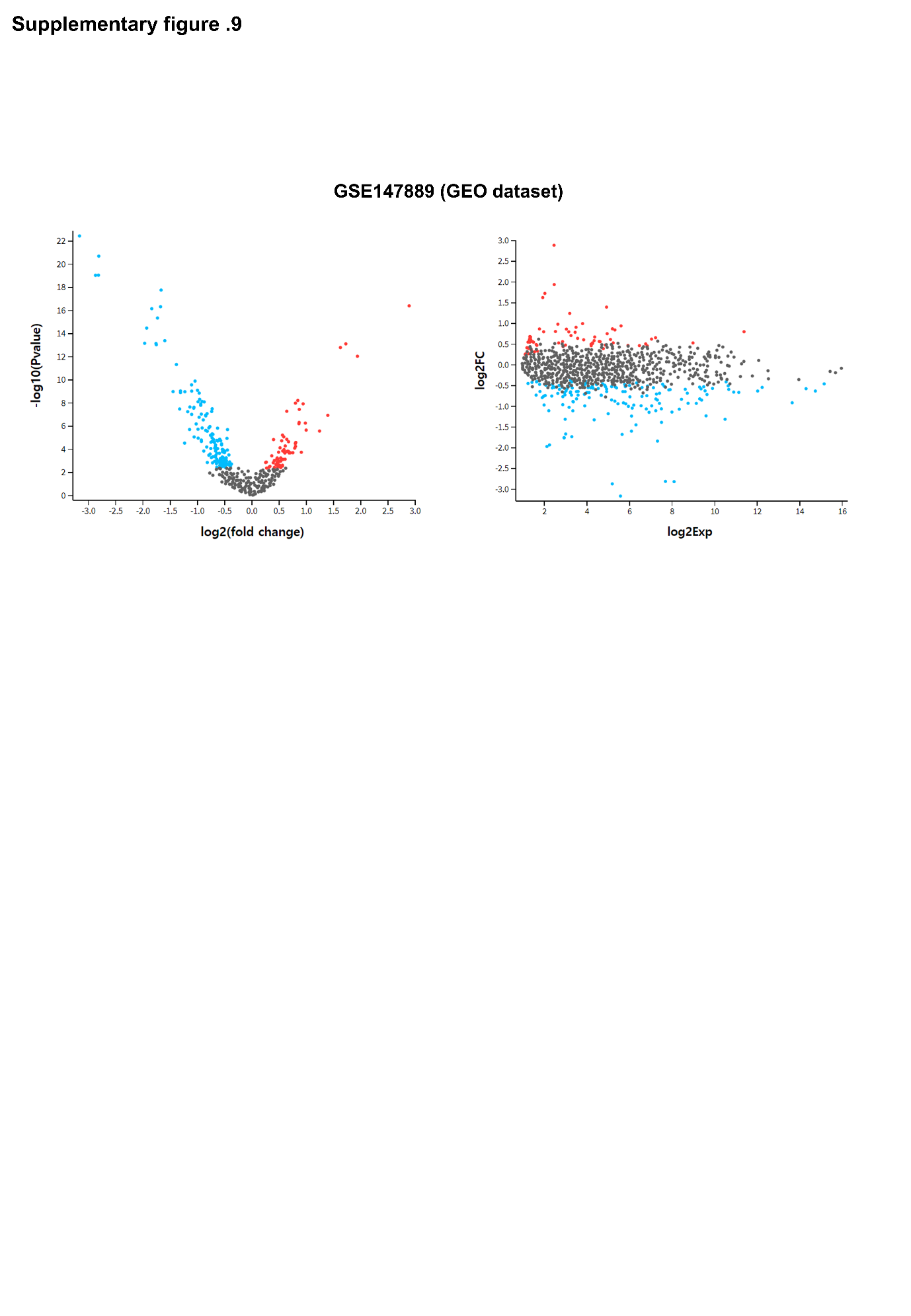
**Supplementary figure 9. Secondary analysis of candidates using the HCC patient database of previous studies**

We analyzed the search for candidates with significant values using volcanic plots and mean-difference plots to investigate promising candidates from this study data and two previous study databases. Data with significant P-value and expression level were displayed in red for positive values and blue for negative values (**GSE147889**).


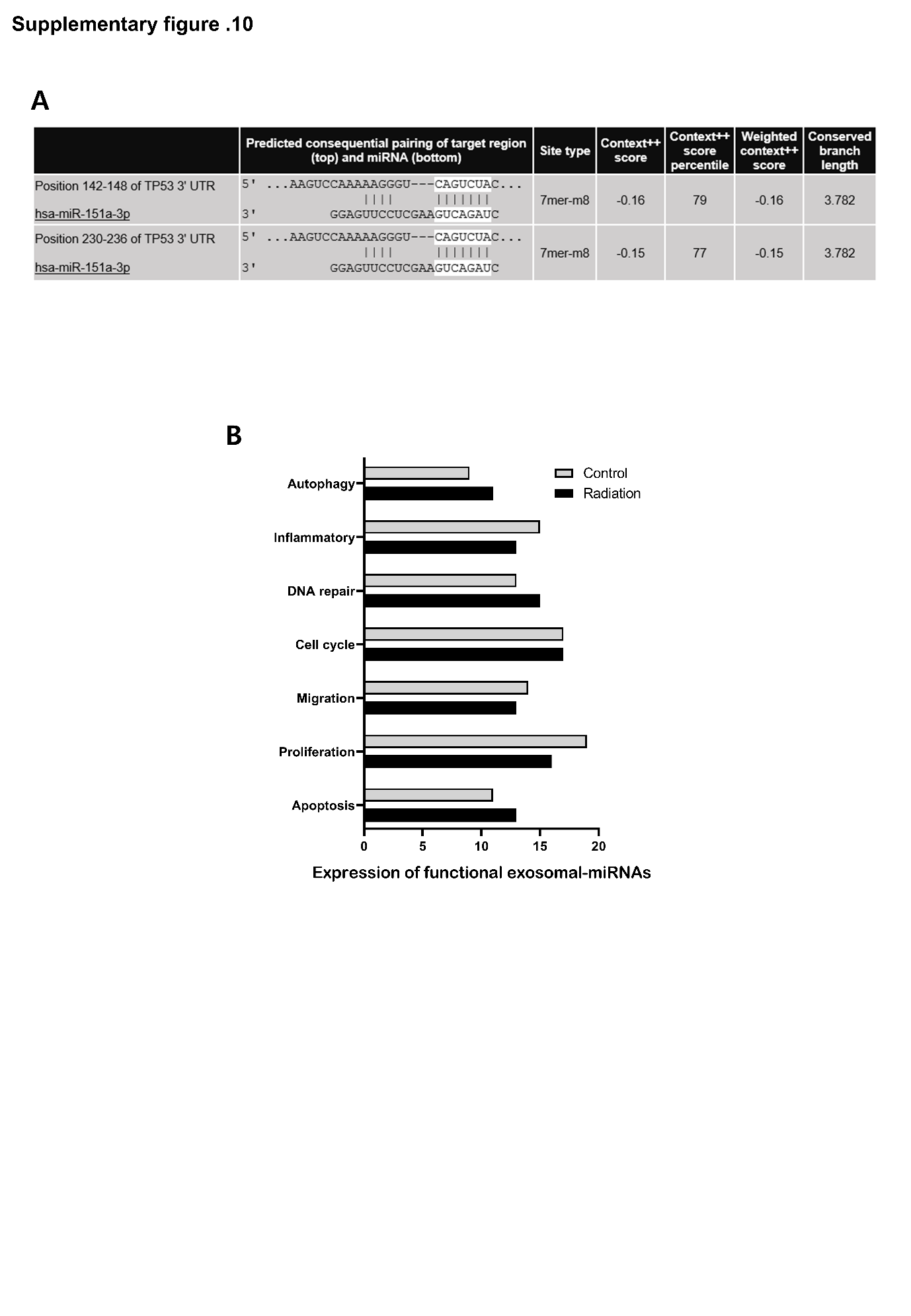
**Supplementary figure 10. Protein target analysis and intracellular function analysis using exosomal miRNA candidates**

Targetscan (http://www.targetscan.org/vert_72/) analysis tool was used to analyze the target protein of each miRNA. As a result of the target analysis of miRNA, it was discovered that miRNA151a-3p can bind to the p53 mRNA target region with a high probability **(A).** The Gene Ontology term was used to discriminate the cellular functions of control and radiation-derived exosomal miRNAs **(B).**

**
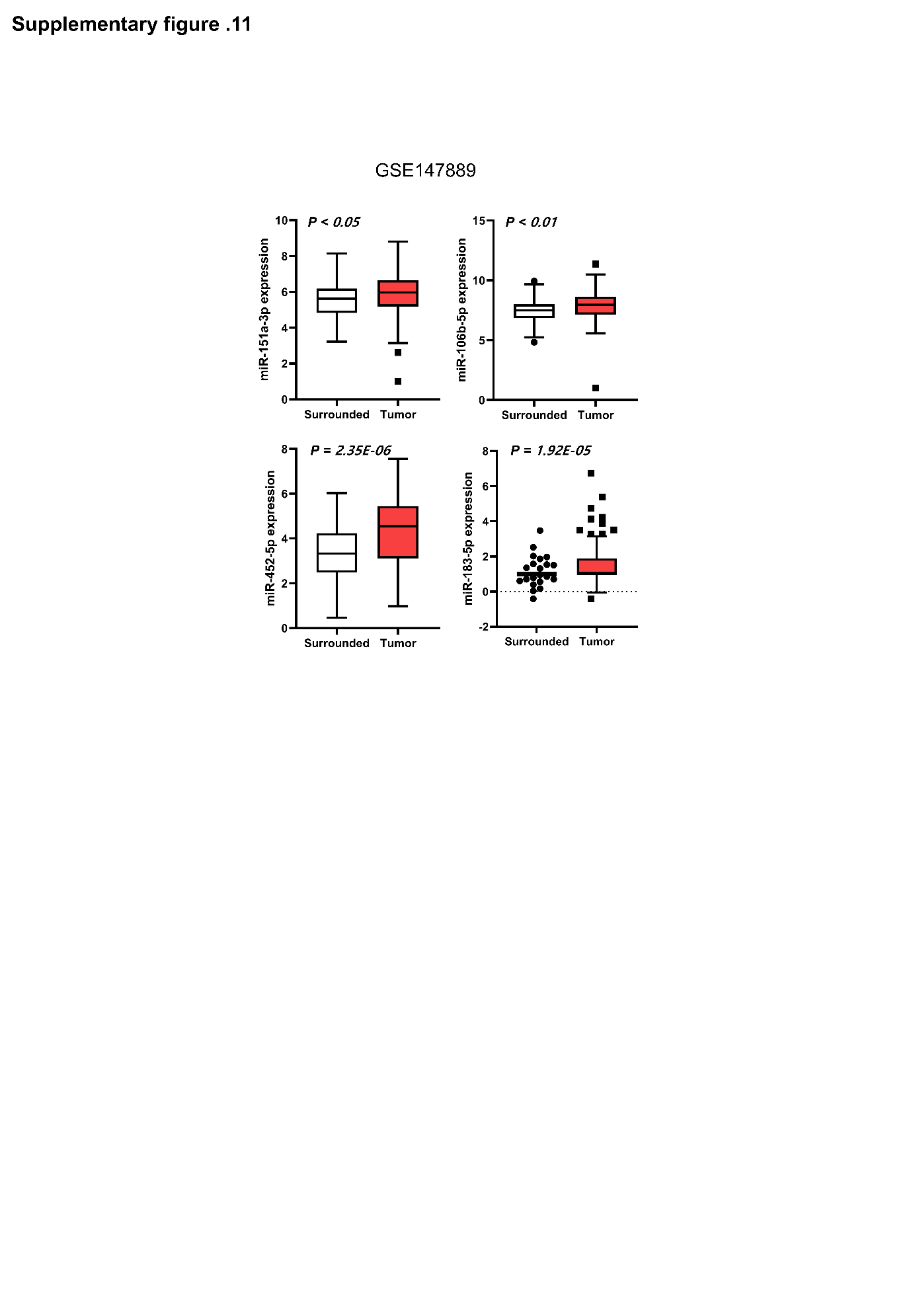
Supplementary figure 11.** **Expression differences in HCC adjacent tissues and tumor tissues of exosomal miRNAs reduced by RT**

Using the GEO dataset (GSE147889), differences in the expression of four exosomal miRNA candidates were investigated in adjacent tissues and tumor tissues of HCC patients. tumor tissues vs. adjacent tissues by Student’s t-test.


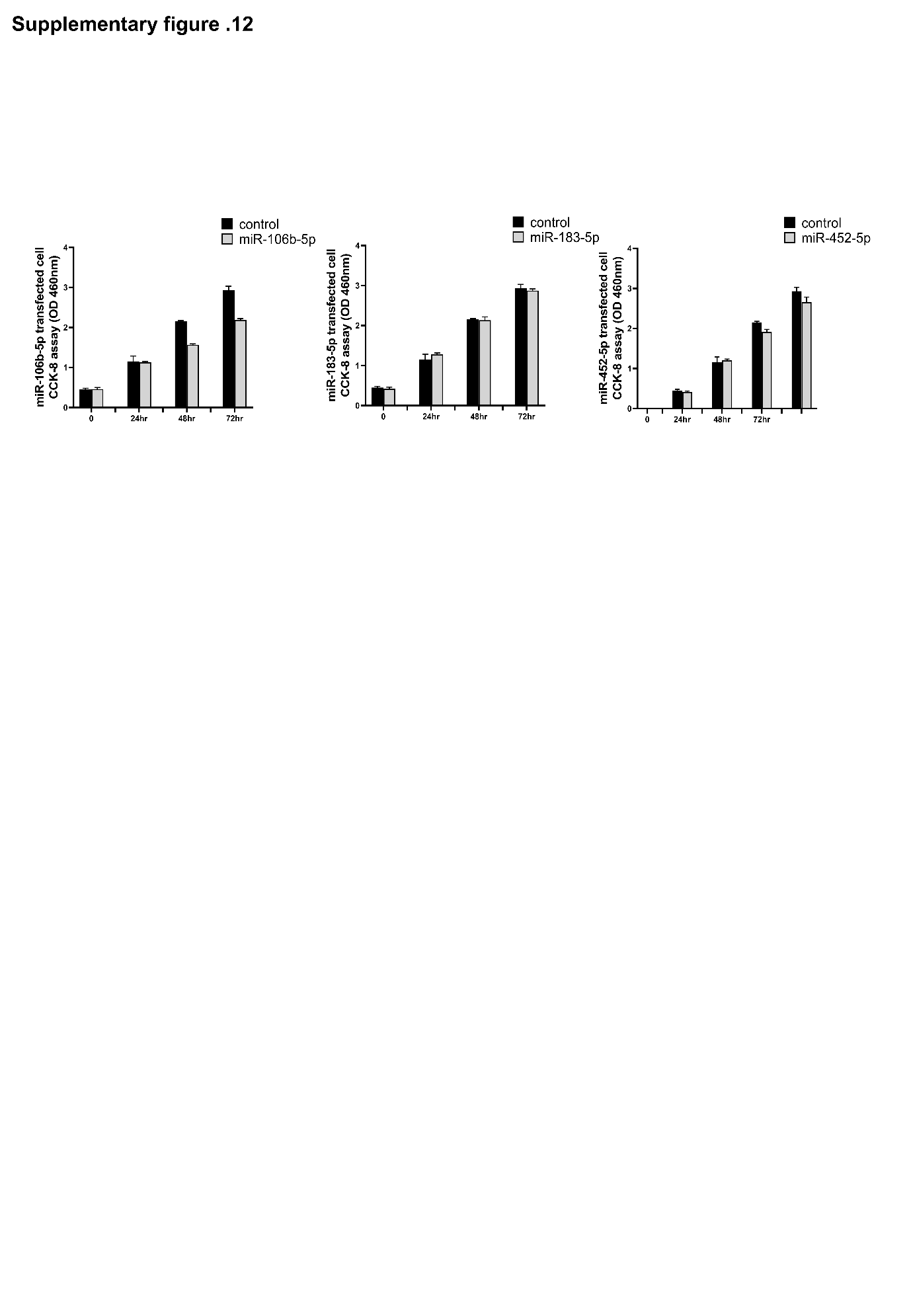
**Supplementary figure 12.** **HCC proliferation assay of reduced miR-106b-5p, miR-183-5p, and miR-452-5p in exosomes after RT.**

HepG2 was transfected with 100 nM of miR-106b-5p, miR-183-5p, and miR-452-5p, respectively, and cell proliferation was investigated at 24-hour intervals up to 72 hours using the CCK-8 assay.


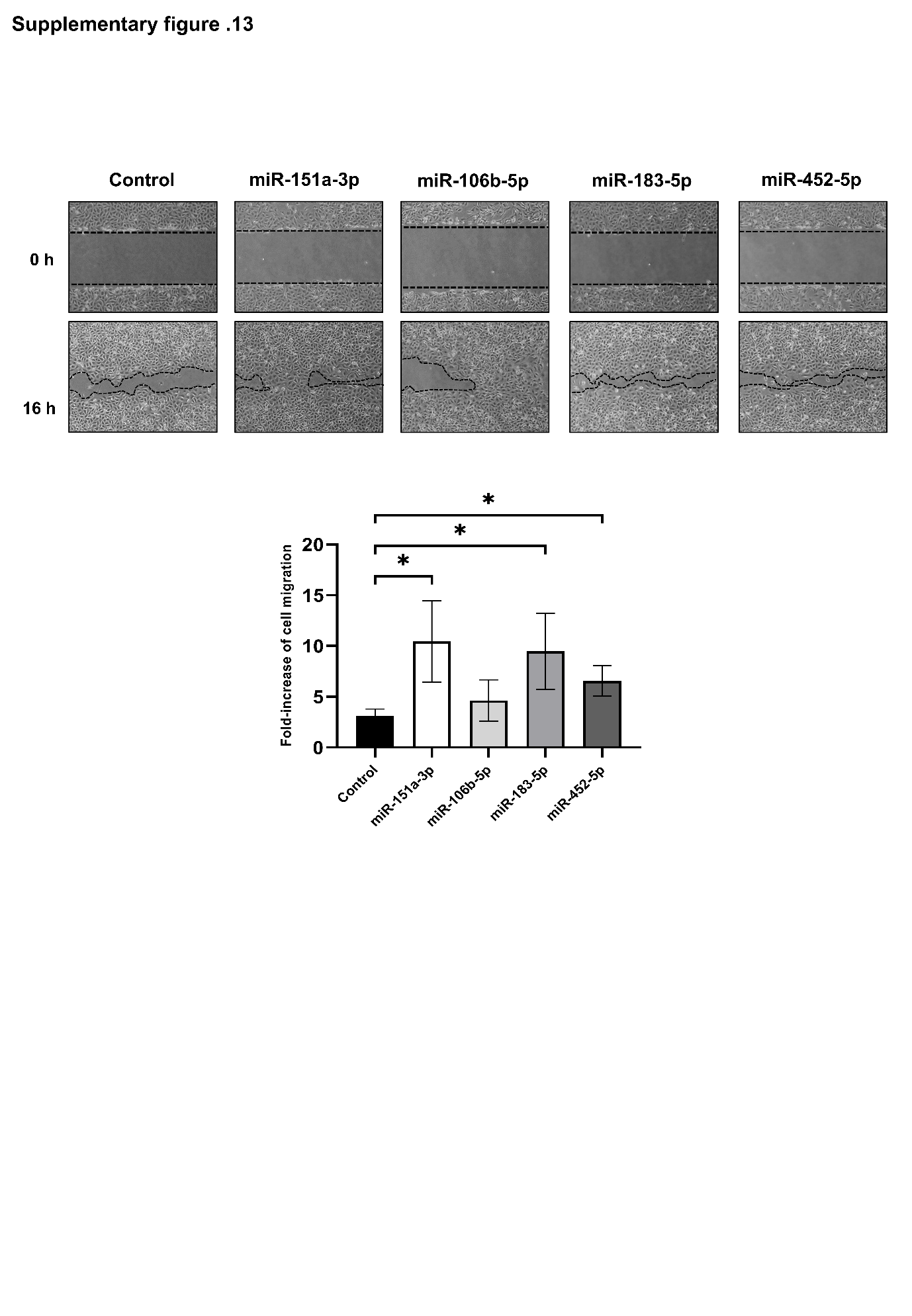

**Supplementary figure 13. Cell migration assay of reduced miR-151a-3p, miR-106b-5p, miR-183-5p, and miR-452-5p in exosomes after RT**

HepG2 was transfected with 100 nM each of miR-151a-3p, miR-106b-5p, miR-183-5p, and miR-452-5p, and a wound healing assay was performed to verify cell migration. Cell culture dishes were performed in at least three separate experiments. Data are shown as mean±SEM. *P < 0.05 vs. control by Student’s t-test.


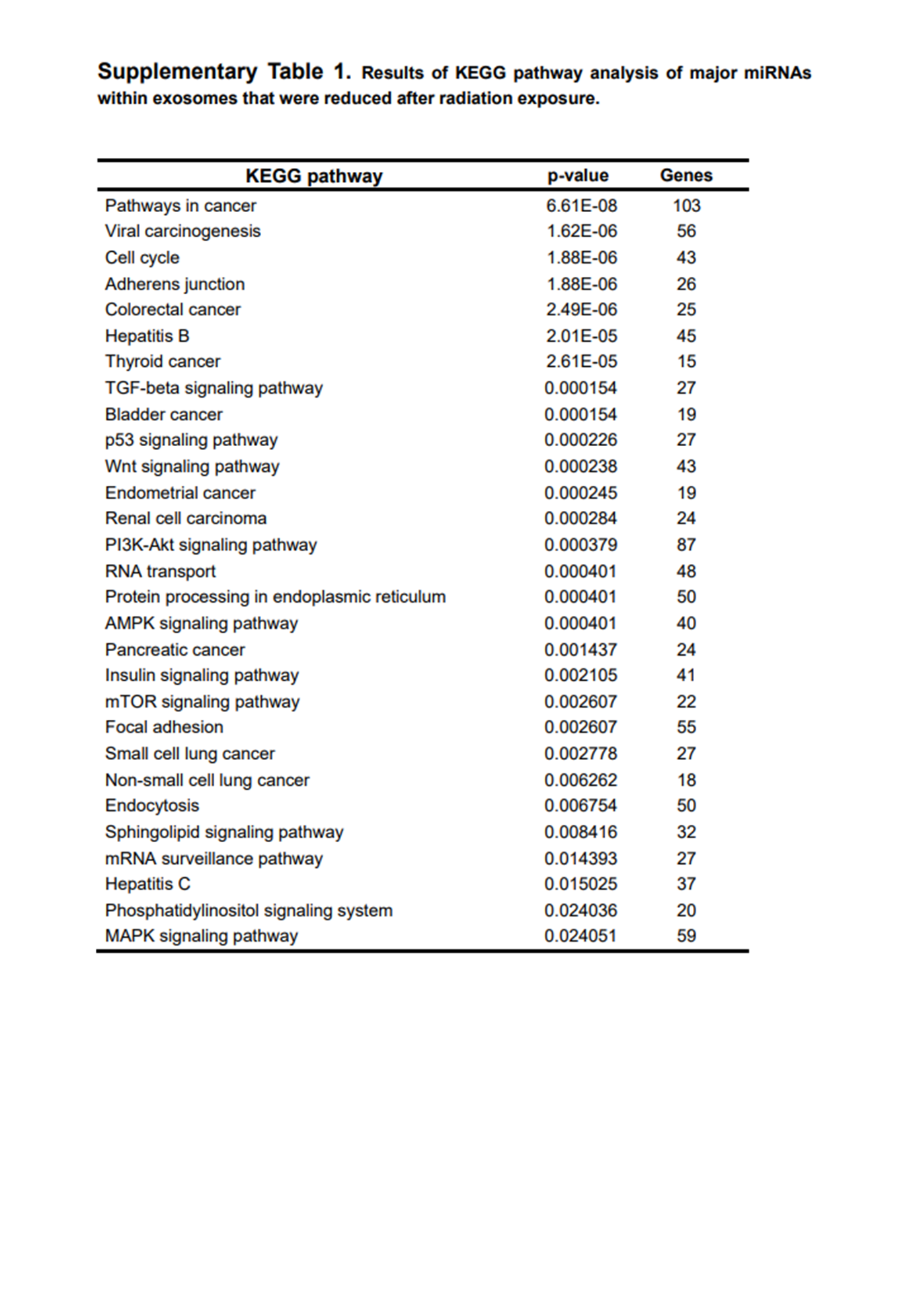


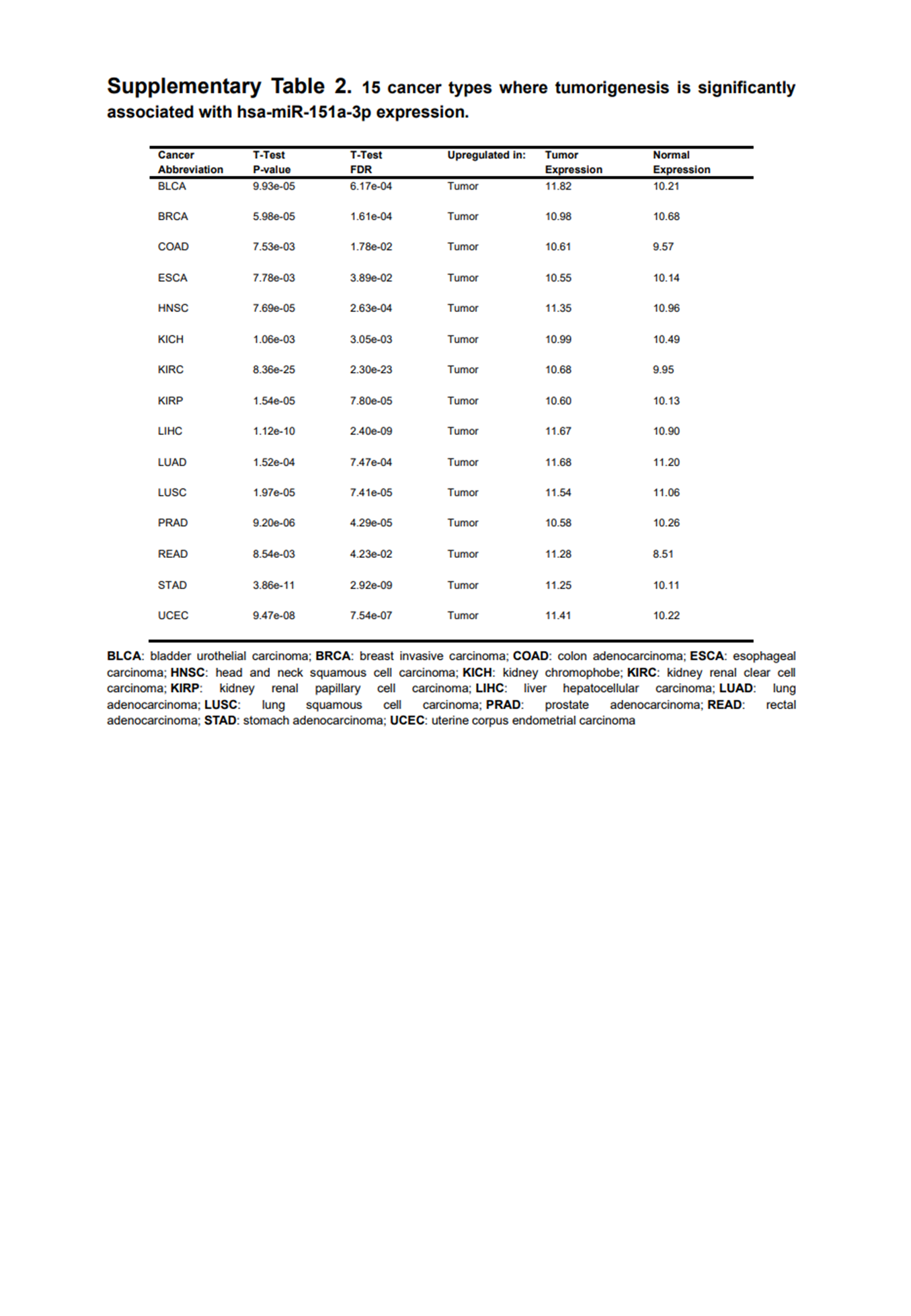

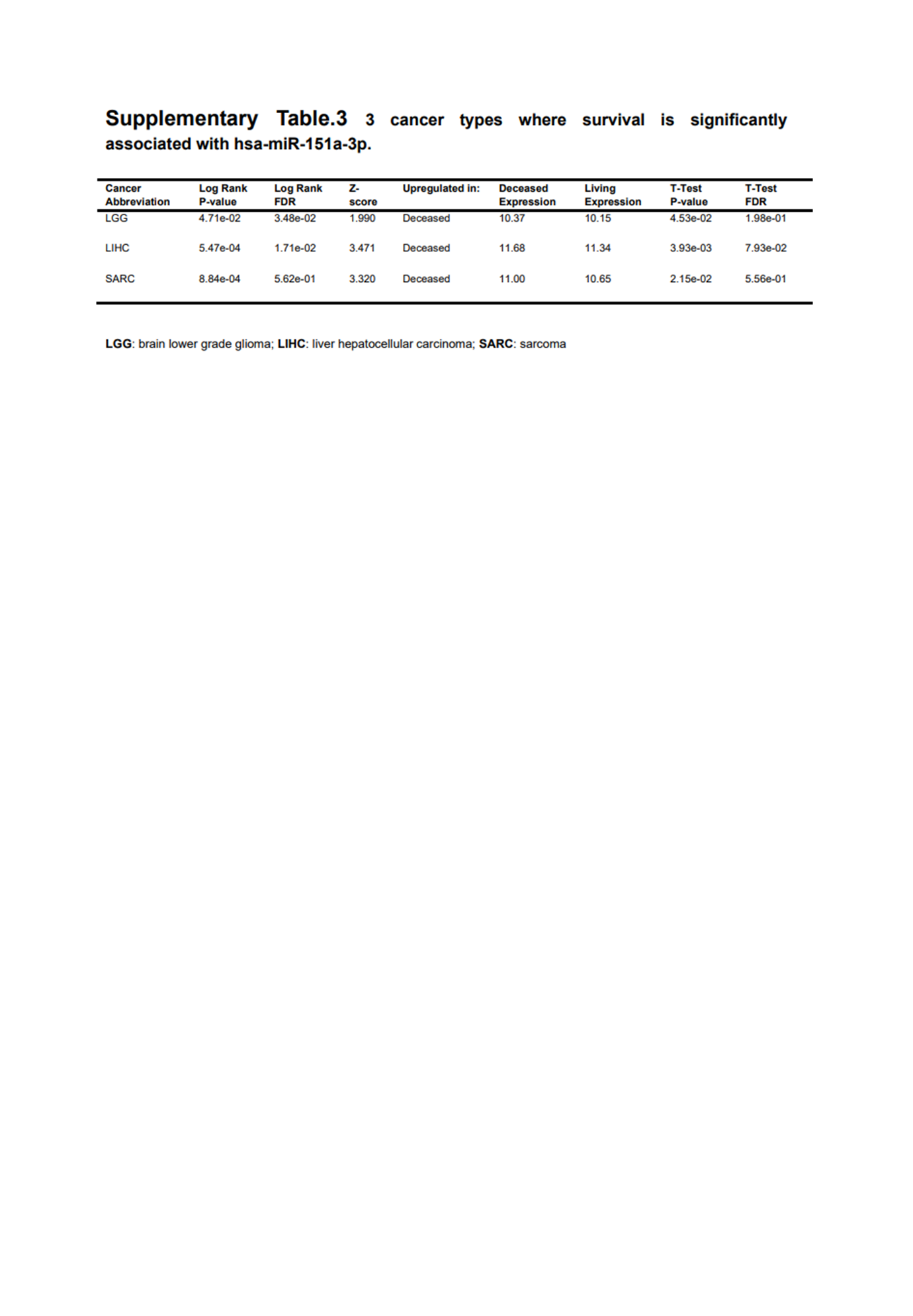

Supplement: Supplementary file 1 — Additional file 1: Supplementary figure 1. Using CRISPR-Cas9 system, a cell line with p53 gene deleted was manufactured in HepG2. We deleted the p53 gene in the p53+/+ HepG2 cell line using the CRISPR-Cas9 system. The target site of Human p53: TGTAACAGTTCCTGCATGGG was investigated by the NGS method. The insertion/deletion frequency was 99.98% (T insertion:19.9%, CCTG deletion:18.8%, AT insertion:19.5%). Supplementary figure 2. Measurement and identification of differences in cell cycle arrest effects according to the presence of the p53 gene in HCC by RT. Changes in cell cycle after irradiation using HepG2, a liver cancer cell line, were measured by FACS. This was done to confirm the radiation resistance effect of conventional p53. Using FACS equipment, it was confirmed that cell cycle arrest was induced in p53+/+, and p53-/-cells by radiation treatment. In p53+/+, powerful cell cycle arrest in the G2/M phase was observed from 6 hours after irradiation and gradually switched to G1 phase cell cycle arrest as time passed. A similar cell cycle alteration was observed in p53-/-cell, but it was verified that the effect was smaller than that of p53+/+cell. Supplementary figure 3. The importance of the P53 gene in RT-induced apoptosis. Changes in apoptosis after irradiation using HepG2, a liver cancer cell line, were measured by FACS. Using FACS equipment, it was confirmed that apoptosis is induced in p53+/+, p53-/-cells by radiation treatment. p53-/-cell had significantly less apoptosis 48 hours after irradiation than p53+/+cell. It showed stronger cell cycle arrest and apoptosis at p53+/+cell compared to p53-/-cell. Supplementary figure 4. RT-induced cellular localization of HDAC5 and p53. To examine shifts in HDAC5 and p53 localization caused by RT, HepG2 was infected with GFP-HDAC5 adenovirus. After exposure to 4 Gy of radiation, time-dependent localization of HDAC5 and p53 was confirmed by immunofluorescence. Supplementary figure 5. Differences in HDAC5 expression in [file 40824_2023_467_MOESM1_ESM.docx]
